# Association between body mass index and prognosis in interstitial lung disease: systematic review and meta-analysis

**DOI:** 10.3389/fmed.2026.1778828

**Published:** 2026-03-17

**Authors:** Yunha Nam, Eun Chong Yoon, Hee-Young Yoon

**Affiliations:** 1 Division of Allergy and Respiratory Medicine, Department of Internal Medicine, Soonchunhyang University Bucheon Hospital, Bucheon, Republic of Korea; 2 Division of Pulmonary and Critical Care Medicine, Department of Internal Medicine, Soonchunhyang University Seoul Hospital, Seoul, Republic of Korea

**Keywords:** body mass index, diffusing capacity, forced vital capacity, interstitial lung disease, mortality, obesity

## Abstract

Interstitial lung disease (ILD) is a heterogeneous group of disorders characterized by lung inflammation and fibrosis. Given inconsistent evidence on the prognostic role of body mass index (BMI), we conducted a systematic review and meta-analysis to investigate the association between BMI and clinical outcomes in ILD. PubMed/MEDLINE, Embase, and the Cochrane Library were searched through May 2024 to investigate the impact of BMI on mortality (24 studies), hospitalization (four studies), baseline lung function (10 studies), and forced vital capacity (FVC) changes (five studies) in ILD patients. BMI was analyzed categorically (obese vs. non-obese) and continuously. Heterogeneity was assessed by subgroup analysis and sensitivity analysis. Twenty-five articles including 23,741 patients were analyzed; more than half were cohort studies. Obesity was associated with lower mortality (RR = 0.91, 95% CI = 0.87–0.94, I^2^ = 0%) and higher BMI was inversely associated with mortality (HR = 0.94, 95% CI = 0.92–0.96, I^2^ = 56%). Obese patients showed lower baseline FVC (MD = -1.61, 95% CI = -3.10 to -0.12, I^2^ = 64%), higher baseline diffusing capacity for carbon monoxide (MD = 1.85, 95% CI = 0.84–2.85, I^2^ = 42%) and a slower FVC decline (MD = 1.26, 95% CI = 0.85–1.68, I^2^ = 0%). Overall, higher BMI may be associated with lower mortality and better lung function in patients with ILD.

**Systematic review registration:**

https://www.crd.york.ac.uk/prospero/, identifier CRD42023461730.

## Background

Interstitial lung disease (ILD) encompasses a diverse group of over 200 chronic lung disorders, characterized by varying degrees of inflammation and fibrosis of the lung interstitium, including idiopathic pulmonary fibrosis (IPF), non-specific interstitial pneumonia, connective tissue disease-ILD (CTD-ILD), hypersensitivity pneumonitis, and cryptogenic organizing pneumonia (1, 2). Despite their varied etiologies, many forms of ILD share pathways involving progressive fibrosis of the lung tissue, leading to impaired gas exchange and respiratory failure (3). The prognosis of ILD varies and is influenced by several factors, such as the specific type of ILD, demographics (e.g., age, sex, and smoking status), symptom severity, lung function, radiographic findings (usual interstitial pneumonia pattern), genetic susceptibility (e.g., short telomerase) and comorbidities (4–7). Consequently, predicting outcomes in patients with ILD is challenging because of this heterogeneity.

Body mass index (BMI) is a measure of body fat based on height and weight. In the general population, BMI exhibits a U-shaped relationship with mortality, with both underweight and overweight conditions associated with increased mortality risk (8, 9). This association underscores the role of BMI as a surrogate marker of overall health status, reflecting the underlying metabolic and inflammatory conditions that significantly affect survival outcomes (10). Although BMI is associated with the prognosis of various health conditions, including cardiovascular diseases, diabetes, and malignancies (11–13), its role in the prognosis of ILD remains unclear. Some studies suggest that a higher BMI may be associated with improved survival in ILD, a phenomenon referred to as “obesity paradox” (14–18). However, other studies have shown that obesity may exacerbate underlying lung diseases by increasing inflammation and mechanical load on the respiratory system, and contribute to comorbidities such as cardiovascular disease or sleep apnea (19, 20). A recent meta-analysis including 18,343 patients with IPF reported that baseline BMI was an independent predictor for low mortality [hazard ratio (HR) = 0.94, 95% confidence interval (CI) = 0.91–0.98], but did not associate with acute exacerbation or hospitalization (21). Forced vital capacity (FVC) and diffusing capacity for carbon monoxide (DLCO) are well-established surrogate markers of disease severity and predictors of prognosis in ILD and are incorporated into validated prognostic models that estimate mortality risk (22). In addition, among patients with ILD, a lower BMI has been reported to be associated with worse physiologic status and/or less favorable lung function trajectories (23). However, the impact of BMI on various outcomes in patients with ILD remains unclear. Therefore, we aimed to identify the association between BMI and the prognosis of ILD, focusing on clinical outcomes, including mortality, hospitalization, as well as physiologic indicators such as lung function parameters.

## Materials and methods

### Search strategy

This systematic review was conducted according to the guidelines outlined in the Preferred Reporting Items for Systematic Reviews and Meta-Analyses statement (24). We performed a thorough literature search using electronic databases including PubMed/MEDLINE, Embase, and the Cochrane Library to identify relevant articles. The search covered articles published from the inception of these databases until May 2024. The search strategy, incorporating terms related to “Interstitial Lung Disease,” “ILD,” “Body Mass Index,” “BMI,” was developed using appropriate keywords, and is detailed in Supplementary Tables 1–3. Furthermore, we included all relevant studies cited in previous comprehensive reviews (21, 25). We manually searched the reference lists of relevant original and review articles to identify eligible studies. The study protocol was registered in PROSPERO (CRD42023461730). The study was exempt from Institutional Review Board approval, as it involved the analysis of published data without human subject involvement or identifiable information.

### Inclusion criteria

The inclusion criteria for the studies were as follows: (1) participants aged ≥ 18 years diagnosed with ILD; (2) BMI measured at baseline or categorized as obese and non-obese groups; (3) outcomes of interest, including clinical endpoints (mortality, hospitalization), longitudinal changes in lung function, and cross-sectional physiologic parameters at baseline (FVC, DLCO); (4) randomized controlled trials, *post hoc* analyses, observational studies (cohort, case-control, or cross-sectional studies); and (5) studies written in English. Cross-sectional studies were included only if they reported baseline lung function outcomes.

The exclusion criteria were as follows: (1) animal or *in vitro* studies; (2) case reports or case series with a small sample size (< 20) due to limited statistical reliability; (3) conference abstracts or posters without full-text availability; and (4) inability to extract data.

When multiple studies were separately reported in a single article, each study was treated as a separate entity.

### Data extraction and quality assessment

The two reviewers (Y. N. and EC. Y.) independently screened titles and abstracts based on predetermined criteria, followed by full-text assessment of eligible studies. Discrepancies were resolved by discussion or consultation with a third reviewer (H-Y. Y.). Data were extracted from full-text articles and Supplementary materials using a standardized approach with a pre-defined Excel form, including study characteristics (author, year, study design, and site), patient demographics, BMI categories and definitions, types of ILD, use of antifibrotic, follow-up duration, and clinical outcomes. For each outcome, we extracted the sample size corresponding to the analytic cohort used in the original study for that specific outcome (e.g., baseline lung function cohort, longitudinal FVC-change cohort, or multivariable time-to-event cohort), rather than the overall registry size, when these differed.

The quality of the included articles was assessed using the Newcastle-Ottawa Scale (NOS), which evaluates selection (4 points), comparability (2 points), and outcome (3 points) for observational studies. *Post hoc* analyses of randomized controlled trials were assessed as observational studies using the NOS, as BMI was not a randomized intervention. Scores > 7 indicated a low risk of bias, scores of 5–7 indicated a moderate risk, and scores < 5 indicated a high risk. Two independent reviewers (Y. N. and EC. Y.) assessed the data with a third-party arbitrator (H-Y. Y.) involved in resolving disagreements and ensuring consensus.

### Definition of obesity

Obese and non-obese groups were categorized based on different BMI criteria in each study using either the World Health Organization (WHO) classification or the WHO Asia-Pacific region definition, depending on the region of the study. Obesity was defined as a BMI of ≥ 30 kg/m^2^ according to the WHO classification, and 25 kg/m^2^ according to the WHO Asia-Pacific region definition (26, 27). When multiple cohorts were included in the study, obesity in each cohort was defined according to region or pre-defined criteria. Participants with BMI values below the defined obesity thresholds were classified as non-obese. Given the use of different BMI cutoffs across regions, subgroup analyses stratified by geographic region (Asia vs. Non-Asian) were performed to account for potential heterogeneity.

### Sensitivity analysis

To assess the robustness of the primary findings, we conducted several additional analyses: (1) pooled estimates were recalculated using the restricted maximum likelihood (REML) estimator instead of the DerSimonian-Laird (DL) method for between-study variance (τ^2^); (2) leave-one-out (LOO) analyses were performed by sequentially removing each study to evaluate the influence of individual studies; (3) analyses were restricted to studies with higher methodological quality (NOS ≥ 5); and (4) for the mortality outcome with BMI as a continuous variable, pooled HRs were restricted to multivariable-adjusted estimates only. All analyses were conducted using random-effects models.

### Exploratory meta-regression analysis

Exploratory univariable meta-regression analyses were performed for mortality with BMI modeled as a continuous variable, given the sufficient number of available studies. Predefined categorical covariates included geographic region (Asian vs. non-Asian), study period (before vs. after 2014), ILD subtype (IPF vs. non-IPF), and antifibrotic use. Continuous covariates included follow-up duration, age, and study quality (NOS score). Regression coefficients (β) were estimated on the log-HR scale, and adjusted R^2^ was used to quantify the percentage of between-study variance explained.

### Statistical analysis

To compare the prognosis between the obese and non-obese groups, the risk ratios (RRs) or HRs with corresponding 95% CIs were calculated for dichotomous outcomes (mortality and hospitalization), and the mean differences (MDs) with 95% CIs were calculated for continuous outcomes (baseline or changes in lung function). In addition, pooled HRs with 95% CIs were used for the meta-analysis of continuous BMI in relation to ILD mortality and hospitalization. When feasible, HRs from multivariate analyses were preferred for the calculations. If multiple subgroups were reported within the obese or non-obese categories, they were combined into a single comparator group by summing sample sizes and calculating the sample-size-weighted mean. The corresponding standard deviation was calculated using the standard approach for combining variances across subgroups, as recommended in the Cochrane Handbook (28). When continuous outcomes were reported as median (range) rather than mean ± SD, the corresponding mean and SD were estimated using the methods described by Wan et al. (29) and Luo et al. (30). When outcomes were reported as mean with 95% CI, SDs were derived from the reported CIs using standard statistical formulas. These conversions were applied only when mean and SD were not directly available. Heterogeneity of the included studies was assessed using the I^2^ statistic. I^2^ values ≤ 30% were considered insignificant, values from 30 to 50% indicated moderate heterogeneity, values from 50 to 75% denoted substantial heterogeneity, and values ≥ 75% indicated considerable heterogeneity (31). A random-effects model was employed to estimate the effect sizes of potential heterogeneity among the studies. Subgroup analyses were performed based on (1) regional comparison between Asian and non-Asian populations; (2) temporal comparison between studies conducted before and after 2014, when antifibrotics were broadly used; (3) comparison based on exposure to antifibrotics at the time of baseline lung function assessment; and (4) comparison between IPF and non-IPF ILD. Publication bias was evaluated using funnel plots and Egger’s regression test for asymmetry when the number of included studies was > 10; these assessments were not performed when fewer studies were available due to limited power (32). In the presence of publication bias, the trim-and-fill method was employed to adjust for missing studies and yield the corrected estimates. Statistical significance was defined as *p* < 0.05. All data analyses were conducted using RevMan software (version 5.4; The Cochrane Collaboration, Copenhagen, Denmark) and R software (version 4.2.2; R Foundation for Statistical Computing, Vienna, Austria).

## Results

### Literature search and study characteristics

**FIGURE 1:**
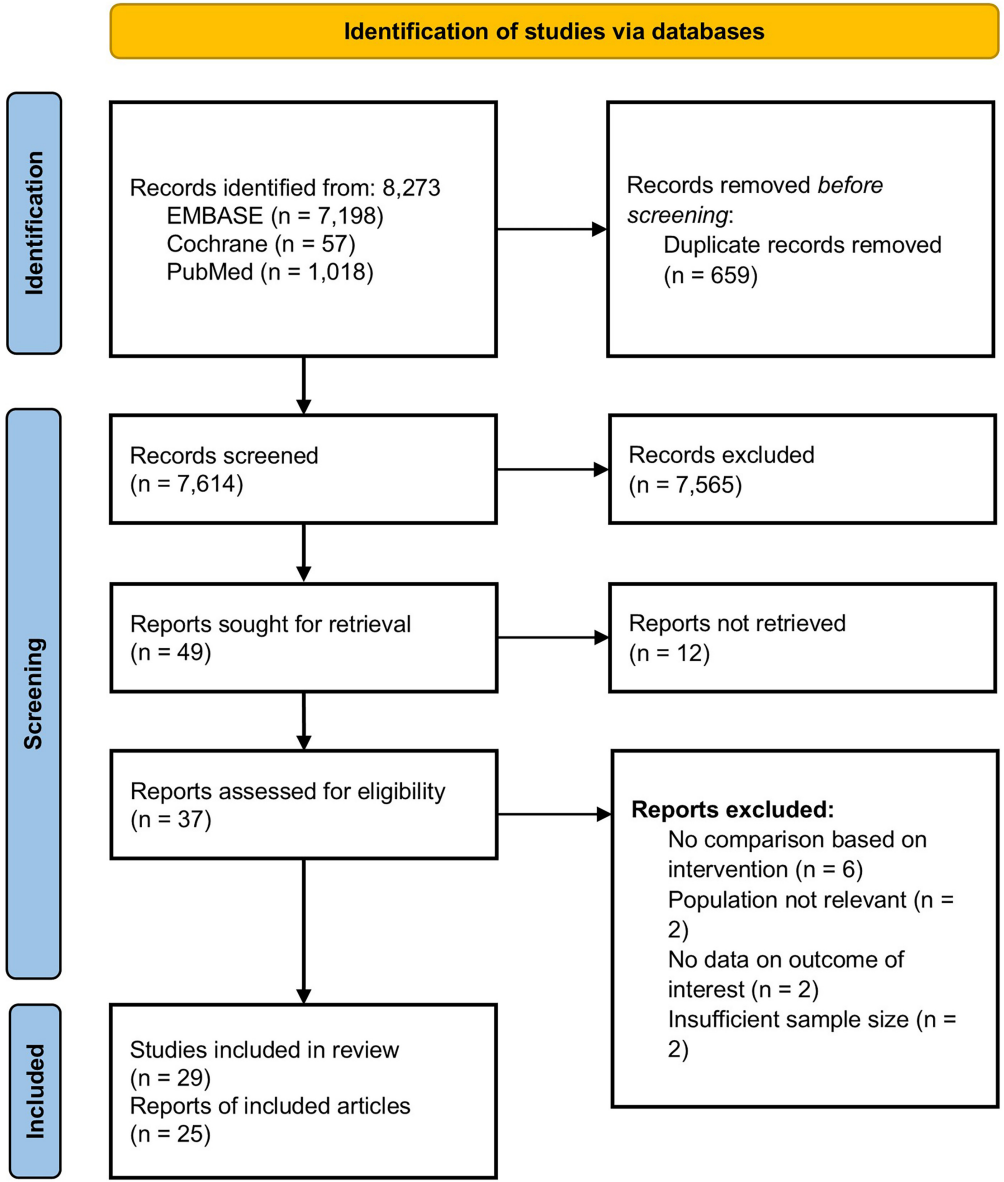
Preferred reporting items for systematic reviews and meta-analyses flow diagram illustrating the study selection process.

A total of 8,273 records were identified during the initial search screening of which 25 full-text articles met the inclusion criteria. In total, 29 studies were included in this analysis (Figure 1). They enrolled a total of 23,741 patients with ILD. Half of the included articles were cohort studies (Table 1). Follow-up periods ranged from a minimum of 12 months to a maximum of 50.3 months. The mean age of the participants ranged from 49 to 76 years, with the proportion of male participants varying from 25.8 to 95.2%. Twenty-four studies (82.8%) were restricted to IPF, while five studies (17.2%) included patients with non-IPF ILD. Among the overall study population, non-IPF ILD comprised CTD-ILD (4.8%), hypersensitivity pneumonitis (2.2%), unclassifiable ILD (3.1%), and pleuroparenchymal fibroelastosis (0.2%). The outcomes analyzed included mortality (24 studies), hospitalization (four studies), FVC (10 studies), DLCO (10 studies), and the rate of FVC decline (five studies). Among 29 studies, 11 studies were divided into groups based on obesity status, and the baseline characteristics of each group are presented in Supplementary Table 4.

**TABLE 1:** Characteristics of the included studies.

Studies		Enrollment period	Number	Design	Follow-up time, month	Outcome	Age	Male (%)	Baseline FVC, % predicted	Baseline DLCO, % predicted
Alakhras et al. (14)		1994–1996	197	Cohort study	NA	Mortality, Lung function	71.4 ± 8.9	70.0	NA	NA
Alhamad et al. (35)		2013–2019	212	Cohort study	NA	Mortality	66.4 ± 11.7	70.7	27.3 ± 20.0	43.0 ± 20.5
Aono (59)		2009–2018	105	Cohort study	NA	Mortality	70 (39–83)	95.2	68.0 (33.6–132.6)	52.6 (21.7–89.9)
Comes et al. (18)	CARE-PF	2016–	1,786	Cohort study	36 (24–72)	Mortality, Lung function	NA	NA	NA	NA
	UCSF	2014–	1,779	Cohort study	24 (12–48)	Mortality, Lung function	NA	NA	NA	NA
Gao et al. (36)		2014–2020	662	Cohort study	NA	Mortality	72.7 ± 7.5	74	71.0 (61.0–85.0)	47.0 (37.0–56.0)
Ikezoe et al. (37)		2010–2014	77	Cohort study	14.4	Mortality	49.0 ± 9.0	62	NA	NA
Jalaber (60)		2016–2018	71	Observational study	23.4	Hospitalization	74.09 ± 7.52	76.1	81.1 ± 17.5	46.0 ± 13.7
Jouneau et al. (33)	Nintedanib	2011–2012	638	*Post hoc* analysis	13	Mortality, Lung function	NA	NA	NA	NA
	Placebo	2011–2012	423	*Post hoc* analysis	13	Mortality, Lung function	NA	NA	NA	NA
Jouneau et al. (38)	Placebo	Ascend 2011–2013 Capacity: 2006–2008 INSPIRE: 2003–2006 RIFF: 2012–2015	1,604	*Post hoc* analysis	12	Lung function	NA	NA	NA	NA
	pirfenidone	ASCEND: 2011–2013 CAPACITY: 2006–2008	623	*Post hoc* analysis	12	Lung function	NA	NA	NA	NA
Jouneau et al. ((38)		2016–2019	153	Cohort study	26 ± 13	Mortality, Hospitalization	72.4 ± 8.1	78	81.7 ± 17.5	45.4 ± 16.8
Kim (61)		2014–2018	1,002	Observational study	23.7	Hospitalization	NA	74.6	NA	NA
Kishaba (62)		2011–2021	39	Observational study	38.6 ± 30.6	Mortality	72.9 ± 7.0	69.2	66.8 ± 14.9	64.9 ± 27.9
Kono (63)		2005–2021	48	Observational study	50.3 ± 33.7	Mortality	65.2 ± 9.7	56.6	68.5 ± 22.9	99.1 ± 28.2
Lee et al. (23)		2016–2018	600	Cohort study	24	Mortality, Lung function	71.0 ± 7.7	74.2	NA	NA
Li (64)		2012–2016	148	Observational study	NA	Mortality	NA	90	NA	NA
Sangani et al. (15)		2015–2019	138	Cohort study	NA	Mortality, Lung function	76.3 ± 9.66	60.1	NA	NA
Snyder (65)		2014–2017	662	Cohort study	30	Mortality	70 (65–75)	74.9	69.6 (60.1–79.9)	41.7 (32.2–50.1)
Suzuki (66)		2000–2015	174	Cohort study	NA	Mortality	69 (64–75)	89.3	80.5 (66.4-92.9)	68.6 (55.4–97.1)
Suzuki et al. (39)		2009–2020	229	Cohort study	NA	Mortality	72.0 (67.5–72.0)	81.2	68.3 (57.0–80.7)	59.0 (44.4–71.3)
Suzuki et al. (40)	Hamamatsu	2009–2019	106	Cohort study	NA	Mortality	72 (68–76)	87.7	65.3 (54.6–76.8)	51 (59.7–64.3)
	Serei	2009–2019	102	Cohort study	NA	Mortality	73 (66–76)	81.4	68.1 (56.5–79.6)	58.5 (45.5–68.5)
Yamaguchi et al. (34)		2008–2021	58	Observational study	NA	Mortality, Lung function	54.6 ± 13.8	25.8	NA	NA
Yamazaki et al. (41)		2008–2017	107	Observational study	NA	Mortality	NA	81.3	NA	NA
Yoon et al. (42)		2002–2018	11,826	Observational study	42 (0-204)	Mortality, Hospitalization	68.9 ± 8.1	73.8	NA	NA
Zinellu et al. (43)		2006–2015	82	Observational study	48	Mortality	72 ± 7	89	77.0 ± 19.2	45.7 ± 18.3
Zinellu et al. (44)		2006–2015	90	Observational study	48	Mortality	70.1 ± 6.3	87.8	74.7 (61.6–89.3)	42.3 (31.0–54.4)

Data are presented as mean ± standard deviation, median (interquartile range), or number (%). FVC, forced vital capacity; DLCO, diffusing capacity for carbon monoxide; CARE-PF, Canadian Registry for Pulmonary Fibrosis; UCSF, ILD registry at the University of California, San Francisco; NA, not available.

### Quality assessment

The results of NOS scoring showed that two articles were of high quality, and 22 articles were considered to be of moderate quality, indicating a potential risk of bias. The median score was 6, with scores ranging from 4 to 9 (Supplementary Table 5).

### Effect of BMI on mortality in ILD

**FIGURE 2:**
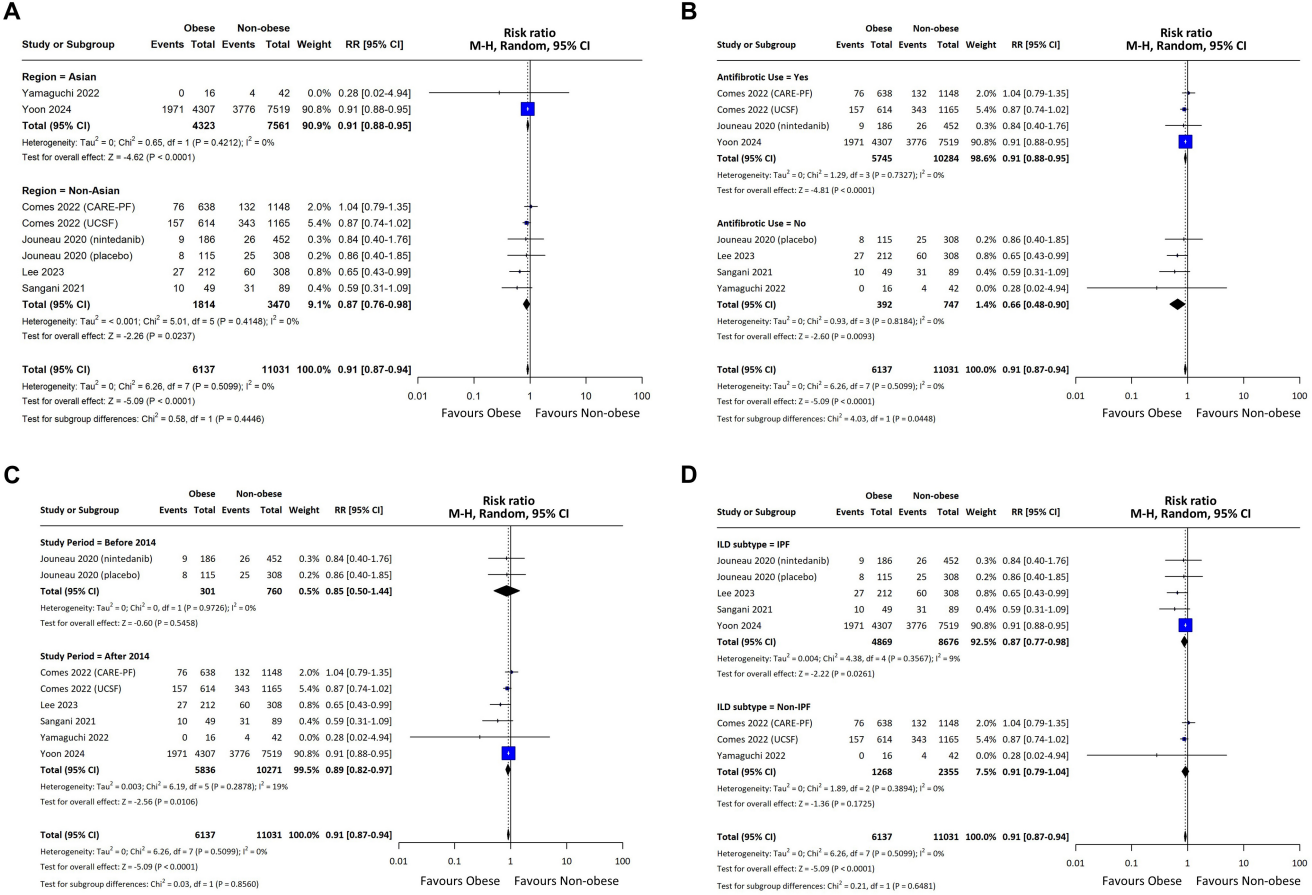
Forest Plot presenting the pooled relative risk for mortality. Obesity defined using region-specific cutoffs (Asia-Pacific ≥ 25; others ≥ 30 kg/m^2^). Subgroups included (A) regional comparison between Asian and Non-Asian populations, (B) comparison based on the use of antifibrotics in studies, (C) temporal comparison between studies conducted before and after 2014, and (D) comparison between IPF and non-IPF ILD. The forest plot displays the individual study results, their respective weights, and the overall combined effect estimate represented by a diamond, with the confidence intervals for each study shown as horizontal lines. CI, confidence interval; M-H, Mantel-Haenszel method; CARE-PF, Canadian Registry for Pulmonary Fibrosis; UCSF, ILD registry at the University of California, San Francisco; IPF, idiopathic pulmonary fibrosis; ILD, interstitial lung disease.

Twenty-four studies reported the mortality rates. Of these, eight provided RR, three provided HR based on BMI categories, and 19 reported HR using continuous BMI. Notably, three studies reported all three types of metrics, which yielded six overlapping counts among the 24 studies. The obese group had lower mortality than the non-obese group (RR = 0.91, 95% CI = 0.87–0.94; *p* < 0.001, I^2^ = 0%) (Figure 2). Subgroup analyses showed that the obese group had lower mortality rates regardless of the region or use of antifibrotic agents (Figures 2A,B). However, a significant association was found only in studies conducted after 2014 (RR = 0.89, 95% CI = 0.82–0.97; *p* = 0.01, I^2^ = 19%), while studies before 2014 showed an RR below 1 but without statistical significance. When analyzing ILD subtypes, a lower risk of mortality was observed in patients with IPF (RR = 0.87, 95% CI = 0.77–0.98; *p* = 0.03, I^2^ = 9%), while non-IPF ILD studies had an RR below 1 without statistical significance. However, the analysis of HR for mortality showed no significant difference between the obese and non-obese groups (Supplementary Figure 1).

**FIGURE 3:**
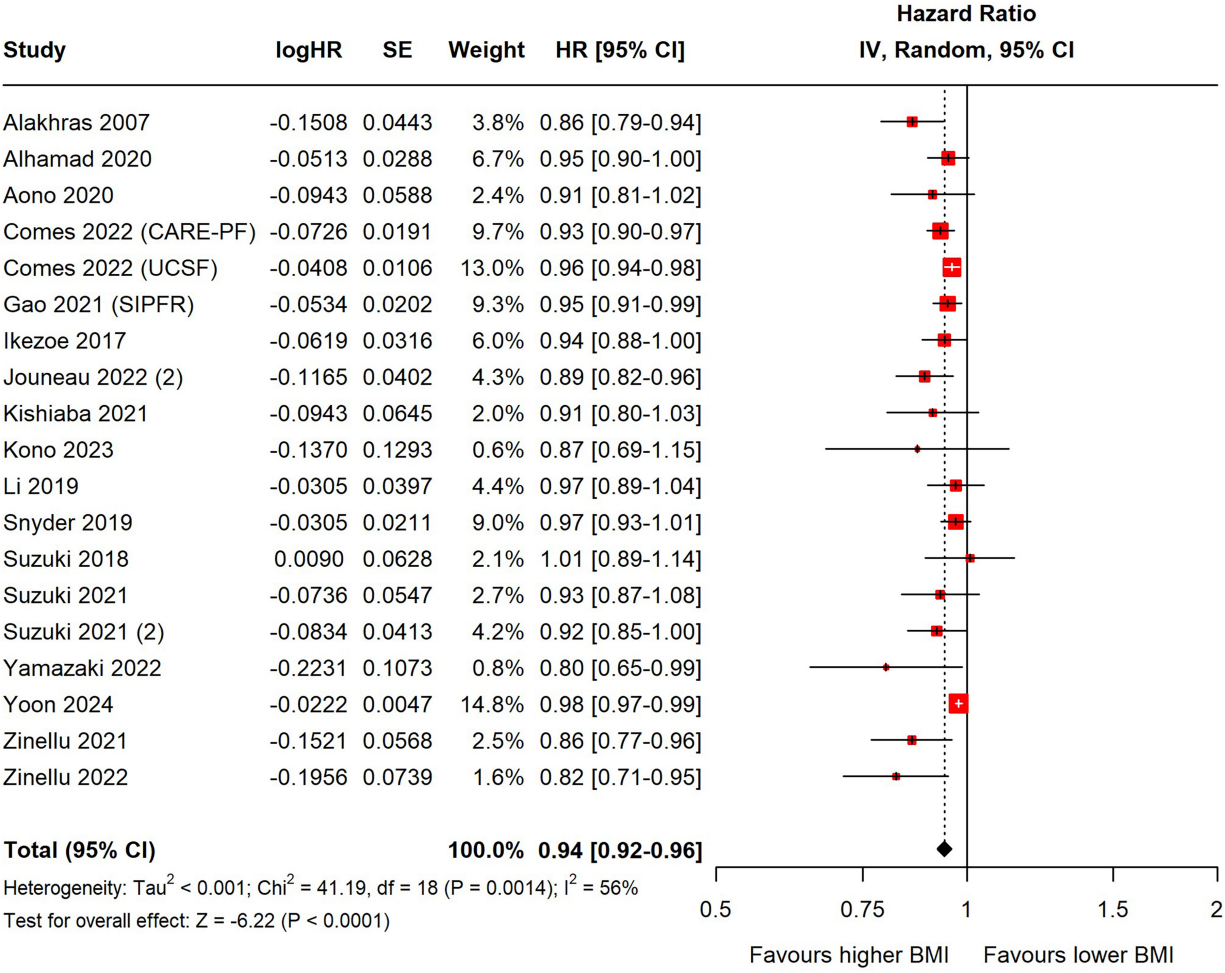
Forest Plot presenting the pooled hazard ratio for mortality. HRs for continuous BMI were interpreted per 1 kg/m^2^ increase. The forest plot displays the individual study results, their respective weights, and the overall combined effect estimate represented by a diamond, with the confidence intervals for each study shown as horizontal lines. BMI, body mass index; SE, standard error; CI, confidence interval; CARE-PF, Canadian Registry for Pulmonary Fibrosis; UCSF, ILD registry at the University of California, San Francisco; SIPFR, Swedish IPF Registry.

Continuous BMI showed a negative association with mortality in patients with ILD (HR = 0.94, 95% CI = 0.92–0.96; *p* < 0.001, I^2^ = 56%), and this association was statistically significant regardless of region, year of diagnosis, use of antifibrotic agents, or ILD subtype (Figure 3 and Supplementary Figures 2A–D).

### Effect of BMI on hospitalization in ILD

In four studies using HRs derived from the univariate Cox regression analysis, lower BMI was identified as a risk factor for hospitalization in patients with ILD (HR = 0.97, 95% CI = 0.95–0.99, *p* = 0.01, I^2^ = 61%) (Supplementary Figure 3A). However, in the analysis using multivariable Cox HRs from three studies, the association reached only marginal statistical significance (HR = 0.98, 95% CI = 0.95–1.00, *p* = 0.08, I^2^ = 74%) (Supplementary Figure 3B).

### Effect of BMI on baseline lung function in ILD

**FIGURE 4:**
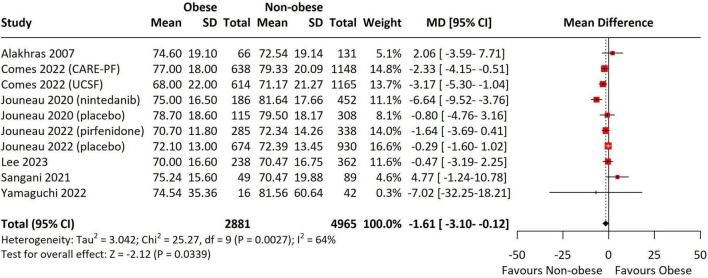
Forest Plot presenting the pooled mean difference for baseline FVC. Obesity defined using region-specific cutoffs (Asia-Pacific ≥ 25; others ≥ 30 kg/m^2^). FVC was pooled as % predicted; studies reporting other units were converted or excluded. The forest plot displays the individual study results, their respective weights, and the overall combined effect estimate represented by a diamond, with the confidence intervals for each study shown as horizontal lines. FVC, forced vital capacity; SD, standard deviation; CI, confidence interval; CARE-PF, Canadian Registry for Pulmonary Fibrosis; UCSF, ILD registry at the University of California, San Francisco.

**FIGURE 5:**
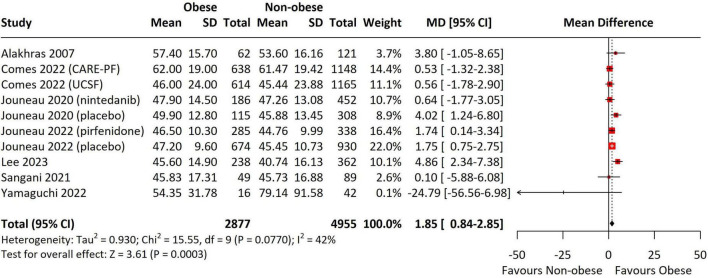
Forest Plot presenting the pooled mean difference for baseline DLCO. Obesity defined using region-specific cutoffs (Asia-Pacific ≥ 25; others ≥ 30 kg/m^2^). DLCO was pooled as % predicted; studies reporting other units were converted or excluded. The forest plot displays the individual study results, their respective weights, and the overall combined effect estimate represented by a diamond, with the confidence intervals for each study shown as horizontal lines. DLCO, diffusing capacity for carbon monoxide; SD, standard deviation; CI, confidence interval; CARE-PF, Canadian Registry for Pulmonary Fibrosis; UCSF, ILD registry at the University of California, San Francisco.

Baseline FVC (studies = 10) was significantly lower in the obese group compared to non-obese groups (MD = -1.61, 95% CI = -3.10 to -0.12, *p* = 0.03, I^2^ = 64%) (Figure 4). In the subgroup analyses, the difference remained significant in the non-Asian studies (MD = -1.59, 95% CI = -3.10 to -0.07, *p* = 0.04, I^2^ = 68%), in studies with antifibrotic use (MD = -2.68, 95% CI = -4.07 to -1.30, *p* < 0.001, I^2^ = 0%), and in non-IPF groups (MD = -2.70, 95% CI = -4.08 to -1.32, *p* < 0.001, I^2^ = 0%) (Supplementary Figures 4A–D). In contrast, analysis of baseline DLCO across 10 studies revealed that obese patients exhibited significantly higher DLCO values (MD = 1.85, 95% CI = 0.84–2.85, *p* < 0.001, I^2^ = 42%) (Figure 5). This significance was observed only in the non-Asian studies (MD = 1.85, 95% CI = 0.92–2.79, *p* < 0.001, I^2^ = 38%), studies not using antifibrotic agents (MD = 2.30, 95% CI = 1.10–3.50, *p* < 0.001, I^2^ = 42%), studies before 2014 (MD = 1.86, 95% CI = 1.10–2.62, *p* < 0.001, I^2^ = 0%), and the IPF groups (MD = 2.30, 95% CI = 1.21–3.38, *p* < 0.001, I^2^ = 36%) (Supplementary Figures 5A–D).

### Effect of BMI on lung function change in ILD

**FIGURE 6:**
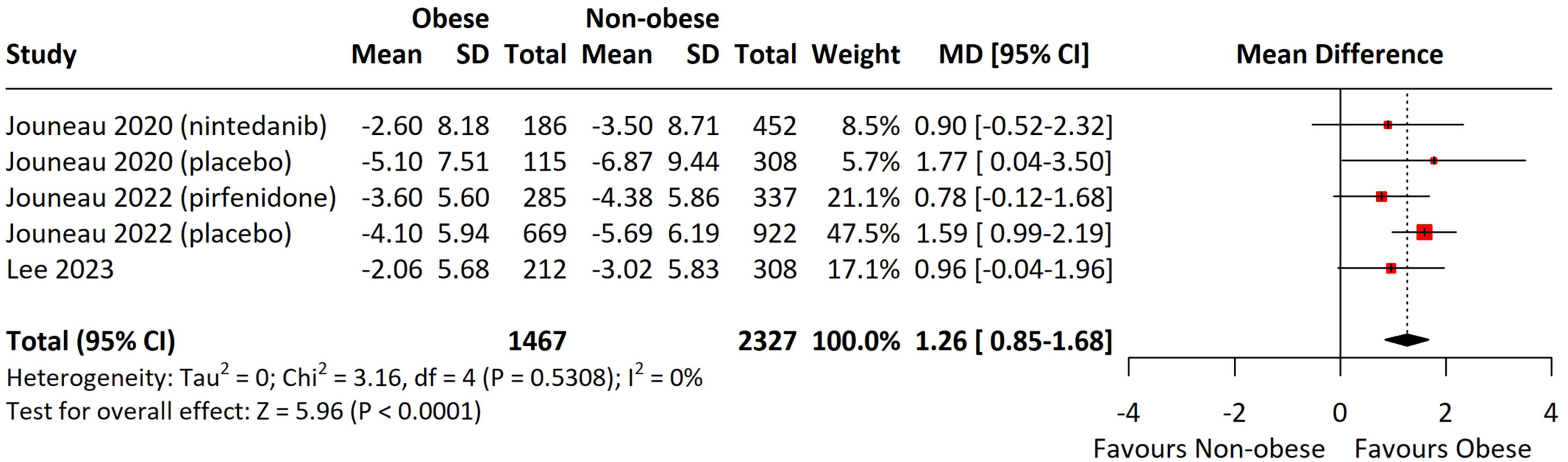
Forest Plot presenting the pooled mean difference for changes in FVC. Obesity defined using region-specific cutoffs (Asia-Pacific ≥ 25; others ≥ 30 kg/m^2^). The forest plot displays the individual study results, their respective weights, and the overall combined effect estimate represented by a diamond, with the confidence intervals for each study shown as horizontal lines. FVC, forced vital capacity; SD, standard deviation; CI, confidence interval.

Changes in FVC were analyzed using data from five studies, all of which reported annual FVC changes (% predicted per year). The obese group exhibited a slower annual decline in FVC compared to the non-obese group (MD = 1.26, 95% CI = 0.85–1.68, *p* < 0.001, I^2^ = 0%) (Figure 6).

### Publication bias

Publication bias for the mortality of BMI as a continuous variable was assessed using a funnel plot and Egger’s regression test, which revealed significant asymmetry and suggested the presence of publication bias (*z* = -5.187, *p* < 0.001). To address this issue, Duval and Tweedie’s Trim and Fill method was applied to estimate seven missing studies on the right side of the funnel plot (Supplementary Figure 6). After adjusting for the missing studies, the meta-analysis results remained consistent with the original findings (HR = 0.95, 95% CI = 0.93–0.98, *p* < 0.001), confirming that publication bias had minimal impact on the overall conclusions. For baseline FVC and DLCO, Egger’s regression tests did not indicate significant funnel plot asymmetry. Trim-and-fill imputed no missing studies for baseline FVC and one study for baseline DLCO; the adjusted pooled estimates for FVC became borderline non-significant (*p* = 0.055), whereas the DLCO association remained significant after adjustment. These findings suggest that potential publication bias is unlikely to materially alter the conclusions, although the baseline FVC result should be interpreted cautiously due to limited precision (Supplementary Table 6).

### Sensitivity analysis

Sensitivity analyses were conducted for each outcome using DL and REML estimators, LOO analyses, and where applicable, restriction to studies with NOS ≥ 5 and multivariable-adjusted estimates (Supplementary Tables 7–13).

For mortality assessed by RR, re-estimation using DL and REML estimators and subsequent LOO analyses showed consistent effect direction with comparable pooled estimates with preserved statistical significance. For mortality analyzed as a continuous BMI variable, DL and REML estimators and LOO analyses produced consistent results. Additional restriction of studies with multivariable-adjusted HRs yielded effect estimates comparable to the primary result. For mortality comparing obese vs. non-obese groups, re-estimation using DL, REML estimators and LOO analyses retained a consistent direction of association, although statistical significance varied depending on the estimator and individual study exclusion.

For hospitalization, univariable HRs were largely unchanged across DL and REML estimators, LOO analyses, and restriction to studies with NOS ≥ 5, which excluded only one lower-quality study (NOS < 5). By contrast, multivariable-adjusted HRs showed attenuation, and statistical significance varied according to the DL or REML estimator and single-study exclusion.

For baseline lung function, the pooled estimate for FVC was sensitive to analytic approach. Although the direction of effect remained consistent, statistical significance varied across DL and REML estimators and LOO analyses. In contrast, pooled estimates for baseline DLCO and FVC change were largely unaffected by estimator choice or individual study exclusion and remained statistically significant throughout.

### Exploratory meta-regression analysis

Exploratory meta-regression analyses for mortality (BMI as a continuous variable) did not identify statistically significant effect modifiers (Supplementary Table 14). Study quality (NOS score) demonstrated a non-significant trend toward explaining between-study heterogeneity (*p* = 0.093).

## Discussion

**FIGURE 7:**
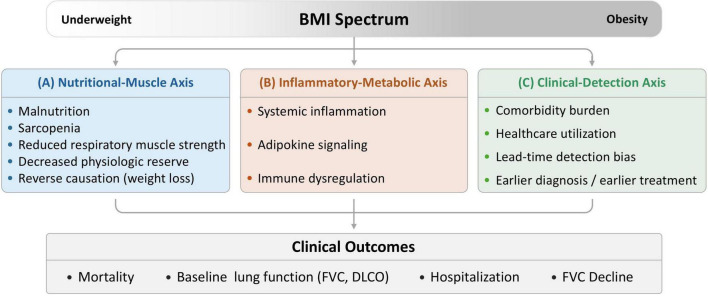
Conceptual framework illustrating potential pathways linking BMI to prognosis in ILD. The schematic outlines three axes–nutritional-muscle, inflammatory-metabolic, and clinical-detection–they may underlie the observed associations between BMI and clinical outcomes (mortality, hospitalization, baseline lung function, and FVC decline). This figure is conceptual and does not imply causality. BMI, body mass index; ILD, interstitial lung disease; FVC, forced vital capacity, DLCO, diffusing capacity for carbon monoxide.

This systematic review and meta-analysis aimed to investigate the association between BMI and the prognosis of patients with ILD. Our findings suggest that BMI significantly affects the clinical outcomes of these patients. Notably, obesity was associated with lower mortality, lower FVC, higher DLCO, and a slower decline in FVC than in the non-obese group. These findings were generally consistent across the different regional groups, regardless of the use of antifibrotic agents. To aid interpretation, Figure 7 summarized plausible mechanisms linking BMI to ILD prognosis within three conceptual axes: nutritional-muscle, inflammatory-metabolic, and clinical-detection pathways.

Our study found that among patients with ILD, those who were obese had a lower mortality rate than non-obese patients and that as BMI increased, the mortality risk decreased. The previous findings support our findings (15, 23). In a rural IPF cohort (*n* = 138), Sangani et al. demonstrated that the obese group (BMI ≥ 30 kg/m^2^) had a lower mortality rate than the non-obese group (BMI < 30 kg/m^2^) (35% vs. 20%, *p* = 0.017). In addition, increasing BMI was associated with improved survival, with mortality rates for BMI categories 25–29.9 kg/m^2^, 20–24.9 kg/m^2^, and < 20 kg/m^2^ being 20, 47, and 75%, respectively (*p* < 0.001) (15). However, in a multicenter USA study, Lee et al. reported no significant difference in mortality rates among the IPF cohort (*n* = 600) when comparing patients with a baseline BMI < 25 kg/m^2^ to those with BMI 25 to < 30 kg/m^2^ (OR = 0.65, 95% CI = 0.34–1.25) or ≥ 30 kg/m^2^ (OR = 0.59, 95% CI = 0.30–1.19) (23). In our study, when applying BMI thresholds of 25 or 30 kg/m^2^ for each region, we consistently found that obese patients had significantly lower mortality rates than non-obese patients, which is in line with previous findings (18, 33, 34). Furthermore, several studies found a significant association between higher BMI and improved survival in ILD (14, 18, 35–44). Our findings further support these associations, as the inverse relationship between BMI and mortality was consistent across different regions, IPF diagnoses, diagnosis years, and antifibrotic treatment status–reinforcing the concept of an “obesity paradox” in patients with ILD, where higher BMI is linked to better outcomes (45).

In the general population, obesity is associated with a worse prognosis due to several factors, including an increased risk of comorbidities such as hypertension, diabetes, cardiovascular diseases, metabolic syndrome, reduced immune function, and chronic inflammation (46). However, in patients with chronic disease including ILD, obese individuals often have a better prognosis (45, 47, 48). This counterintuitive finding may be attributed to several factors, including better overall health status, greater muscle mass, and energy stores, which may help patients cope with the physical demands of their condition (49, 50).

In contrast, underweight patients with ILD often experience disease progression that leads to reduced oral intake, subsequent weight loss, and malnutrition, which are associated with more severe disease and worse prognosis (51, 52). In contrast, obese patients tend to have a higher prevalence of comorbidities, which increases the frequency of healthcare visits and may result in incidental ILD detection before symptoms develop (15). Early detection can create a “lead-time bias,” where earlier diagnosis results in prolonged survival without altering the disease course (53, 54). A single-center cohort study on IPF (*n* = 138) supported these findings, showing that the obese group (BMI ≥ 30 kg/m^2^) was younger and had higher rates of comorbidities than the non-obese group (BMI < 30 kg/m^2^) (15).

According to our results, the obese group showed a slower decline in FVC compared with the non-obese group in patients with IPF, which was similar to the previous findings (23, 33, 55). In a *post hoc* analysis of the INPULSIS trial, regardless of nintedanib treatment, patients with IPF with a BMI ≥ 30 kg/m^2^ had a less pronounced decline in FVC over 52 weeks than those with a BMI < 25 kg/m^2^ (33). Similarly, the CAPACITY trial demonstrated a comparable trend over 1 year, independent of pirfenidone treatment (55). Lee et al. also found that patients with IPF with a baseline BMI < 25 kg/m^2^ had a significantly greater estimated annualized decline in FVC compared with those with a baseline BMI ≥ 30 kg/m^2^, difference of 1.47% predicted per year (95% CI = 0.01–2.93) over 24 months follow-up (*n* = 600) (23). Although the exact mechanisms underlying these findings remain unclear, one possible explanation is that BMI may partially reflect a preserved nutritional or functional status, including muscle mass. In patients with ILD, better physical condition and participation in pulmonary rehabilitation have been associated with improved exercise capacity and quality of life (56). Consequently, a higher BMI may reflect relatively preserved nutritional or functional status rather than being directly protective. However, further research is required to better understand the specific pathways through which BMI and muscle mass affect lung function in patients with ILD.

In our study, DLCO was higher and FVC was lower in the obese group than in the non-obese group. The reduced FVC in obese patients could be attributed to mechanical limitations in lung expansion, increased airway resistance, and altered respiratory muscle function associated with obesity (57). In contrast, the higher DLCO observed in obese patients may be related to increased pulmonary blood volume, which can enhance pulmonary capillary blood volume and subsequently increase DLCO (58). These findings suggest that obesity has a complex effect on lung function in patients with ILD, potentially improving parameters such as DLCO while impairing others such as FVC. Importantly, the reduction in FVC may reflect obesity-related mechanical constraints rather than more advanced fibrotic disease severity.

Our study had several limitations. First, all the included studies were retrospective observational cohorts, limiting causal inferences and potentially introducing confounding factors. Although sensitivity analyses were conducted stratifying factors by region, antifibrotic agent use, and timing of antifibrotic introduction, some biases may remain. Importantly, obesity was defined using region-specific BMI cutoffs (≥ 30 kg/m^2^ in non-Asian studies vs. ≥ 25 kg/m^2^ in Asian studies), which may introduce clinical and methodological heterogeneity and limit direct comparability of pooled categorical estimates. Although regional subgroup analyses were performed, the small number of Asian studies restricts firm conclusions regarding potential regional differences. Second, the heterogeneity of ILD subtypes among the participants could have influenced the results. To address this, we stratified our analyses into IPF and non-IPF groups, which yielded similar results. However, the small sample size for each ILD subtype prevented further subgroup analyses. Moreover, because approximately 90% of the overall study population comprised patients with IPF, pooled estimates are largely driven by IPF data. Therefore, caution is warranted when generalizing these findings to the broader ILD population, particularly for less common non-IPF ILD subtypes. Further analyses using specific ILD subtypes are necessary to better understand the effects within each group. Third, potential confounders, such as smoking status, lung function, physical activity levels, and other comorbidities, were not fully accounted for. To minimize this impact, we included multivariable HRs in our meta-analysis whenever possible. Fourth, because most included studies were judged to have a moderate risk of bias, residual confounding and selection bias may have influenced the magnitude of the pooled association. Although sensitivity analyses yielded comparable results, conclusion should be interpreted with caution. Finally, our analysis exhibited substantial heterogeneity across the included studies. Although we applied a random-effects model to account for variability, residual heterogeneity may have affected the robustness of our findings. Especially, in the hospitalization analysis, we limited inclusion to studies reporting multivariable-adjusted estimates; however, the covariates included in these models varied across studies, which likely contributed to the observed heterogeneity and necessitates cautious interpretation. We therefore conducted a LOO sensitivity analysis, which demonstrated that the pooled estimate for hospitalization was influenced by the inclusion of individual studies. Despite these limitations, our study provides valuable insights into the relationship between BMI and prognosis in patients with ILD by utilizing a meta-analysis to highlight the importance of considering BMI when managing ILD.

## Conclusion

In conclusion, this meta-analysis demonstrates that a higher BMI in patients with ILD is associated with lower mortality and a slower decline in lung function. These findings suggest that BMI may serve as a readily available marker to support clinical risk assessment, particularly in identifying patients with low physiologic reserve who may benefit from closer monitoring or nutritional evaluation. However, BMI should be interpreted as a marker of overall health status rather than a direct therapeutic target. Future research should focus on prospective longitudinal studies that account for various confounding factors to clarify the mechanisms underlying these associations.

## Data Availability

The raw data supporting the conclusions of this article will be made available by the authors, without undue reservation.
