# Supplementary material for: Association between body mass index and prognosis in interstitial lung disease: systematic review and meta-analysis
Source: Front Med (Lausanne). 2026 Mar 17;13:1778828. doi: 10.3389/fmed.2026.1778828 (PMC13036216; doi:10.3389/fmed.2026.1778828)
Supplement: Supplementary file 1 [file Data_Sheet_1.docx]

**Additional file**

**Supplementary Figure 1** Forest Plot presenting the pooled hazard ratio for mortality by obesity status.


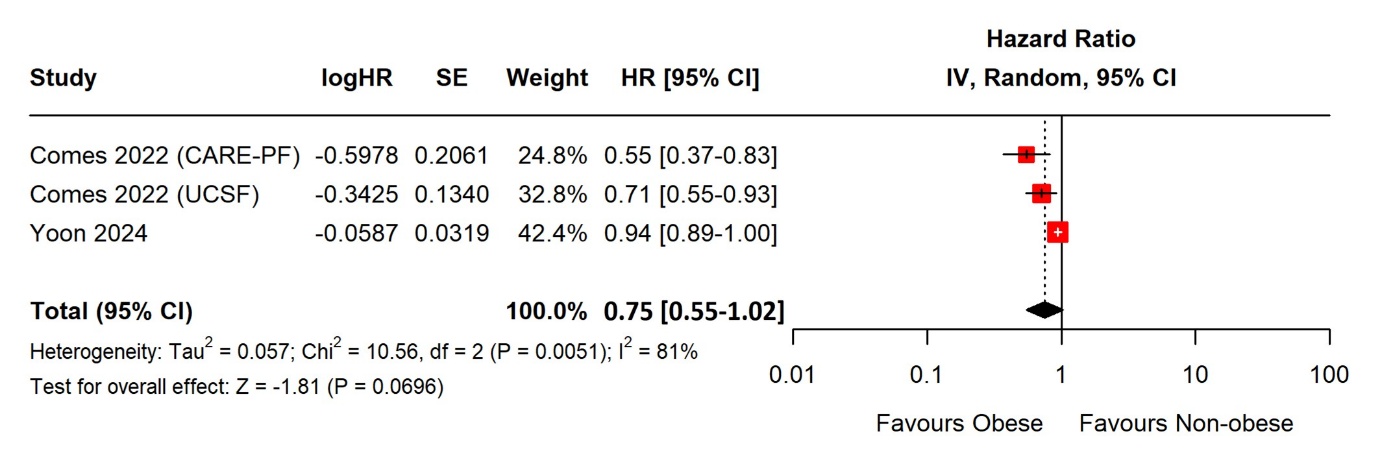


Obesity defined using region-specific cutoffs (Asia-Pacific ≥25; others ≥30 kg/m²).

The forest plot displays the individual study results, their respective weights, and the overall combined effect estimate represented by a diamond, with the confidence intervals for each study shown as horizontal lines.

Abbreviations: SE, standard error; CI, confidence interval; CARE-PF, Canadian Registry for Pulmonary Fibrosis; UCSF, ILD registry at the University of California, San Francisco; HR, hazard ratio; BMI, body mass index.

**Supplementary Figure 2** Forest Plot presenting the pooled hazard ratio for mortality.


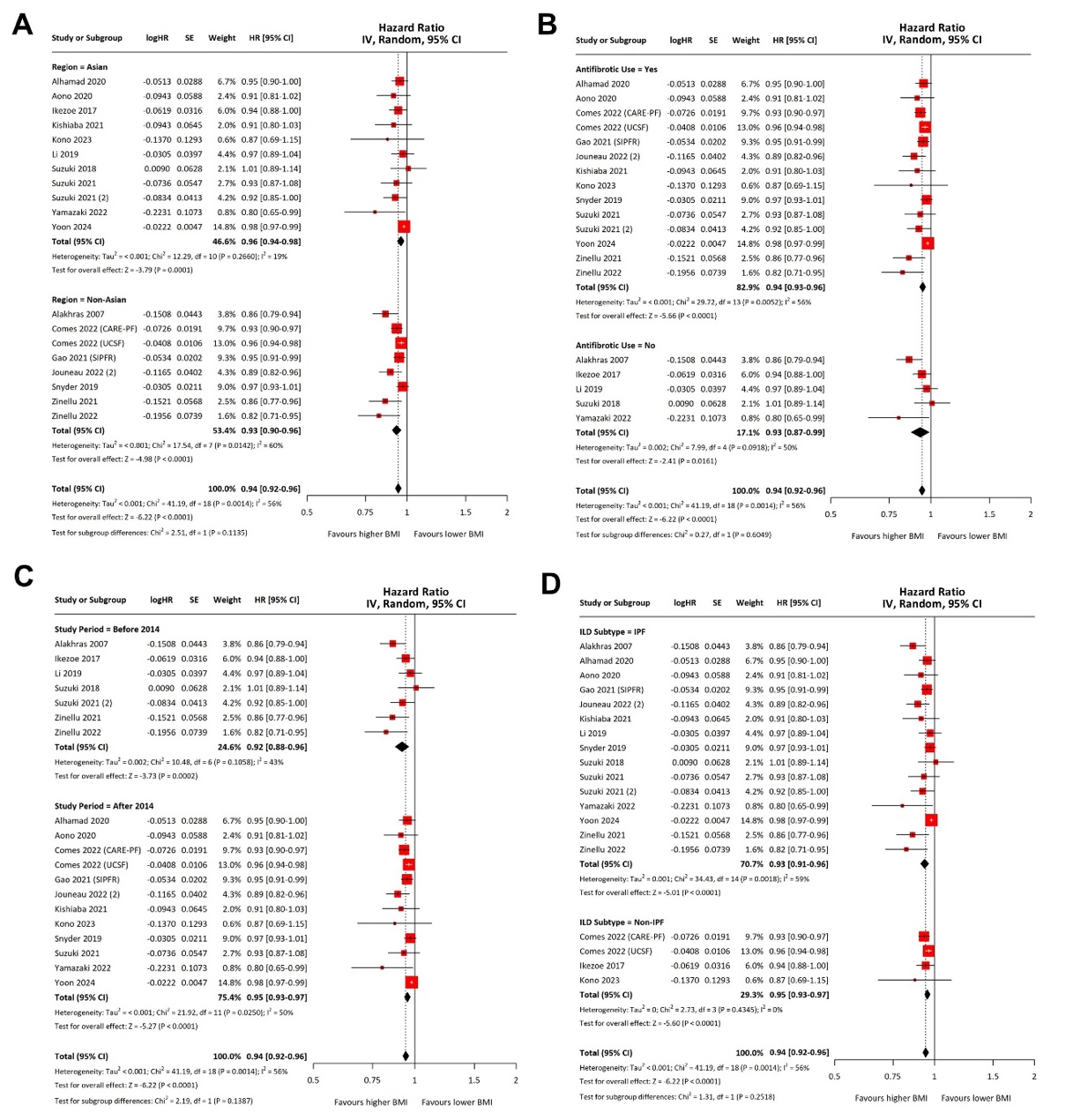


HRs for continuous BMI were interpreted per 1kg/m^2^ increase.

Subgroups included (A) regional comparison between Asian and Non-Asian populations, (B) comparison based on the use of antifibrotics in studies, (C) temporal comparison between studies conducted before and after 2014, and (D) comparison between IPF and Non-IPF ILD.

The forest plot displays the individual study results, their respective weights, and the overall combined effect estimate represented by a diamond, with the confidence intervals for each study shown as horizontal lines.

Abbreviations: SE, standard error; CI, confidence interval; CARE-PF, Canadian Registry for Pulmonary Fibrosis; UCSF, ILD registry at the University of California, San Francisco; SIPFR, Swedish IPF Registry; BMI, body mass index; HR, hazard ratio; IPF, idiopathic pulmonary fibrosis; ILD, interstitial lung disease.

**Supplementary Figure 3** Forest Plot presenting the pooled hazard ratio for hospitalization.

A. Univariable hazard ratio


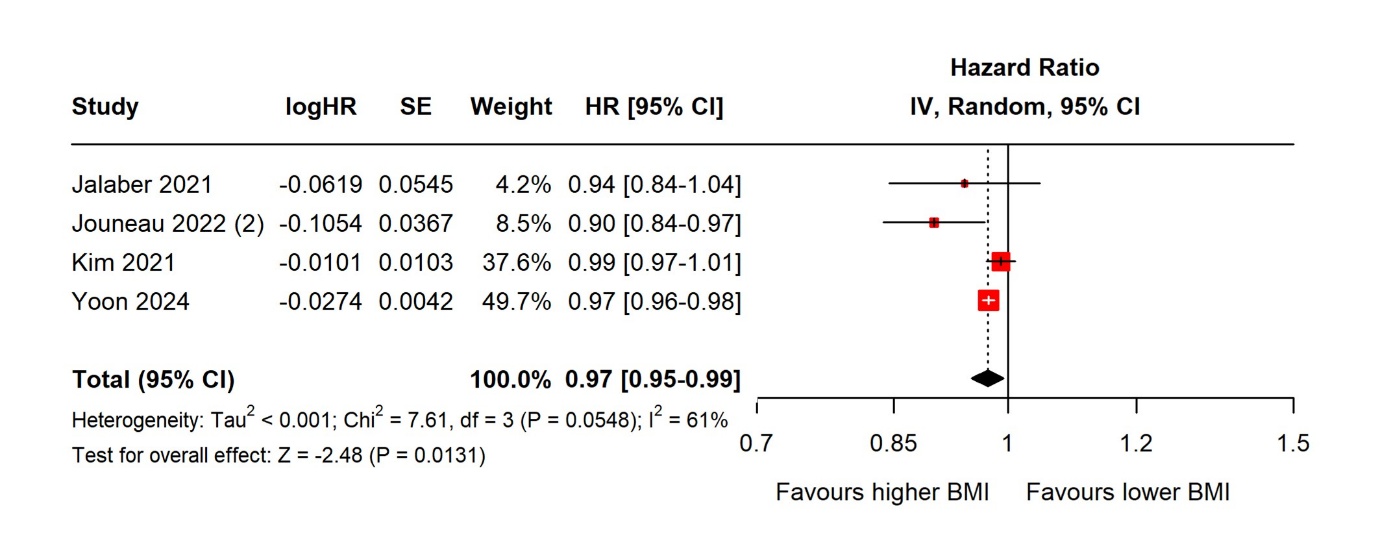


B. Multivariable hazard ratio


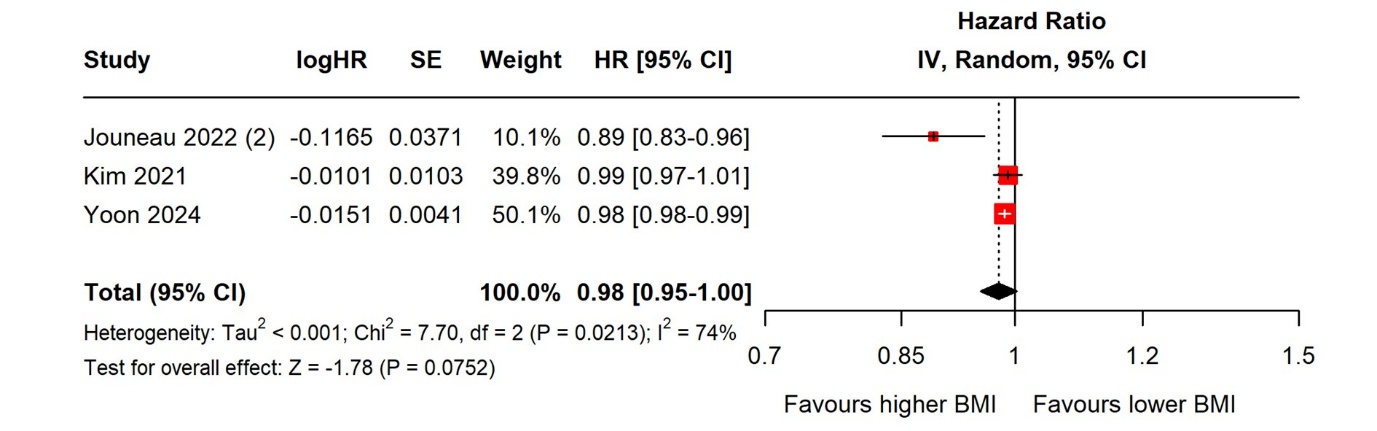


HRs for continuous BMI were interpreted per 1kg/m^2^ increase.

The forest plot displays the individual study results, their respective weights, and the overall combined effect estimate represented by a diamond, with the confidence intervals for each study shown as horizontal lines.

Abbreviations: SE, standard error; CI, confidence interval; BMI, body mass index; HR, hazard ratio.

**Supplementary Figure 4** Forest Plot presenting the pooled mean difference for baseline FVC.


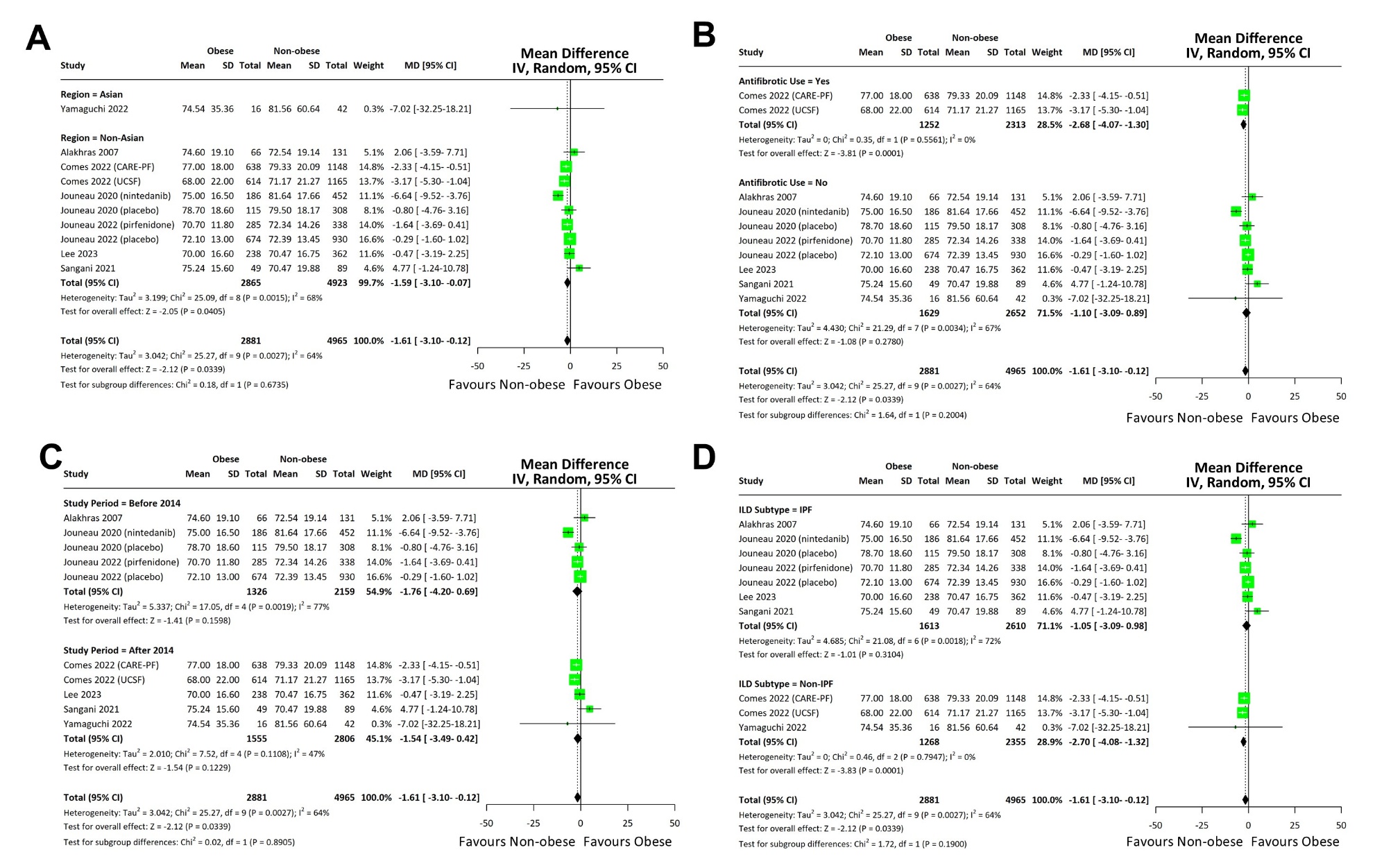


Obesity defined using region-specific cutoffs (Asia-Pacific ≥25; others ≥30 kg/m²).

FVC was pooled as % predicted; studies reporting other units were converted or excluded.

Subgroups included (A) regional comparison between Asian and Non-Asian populations, (B) comparison based on the use of antifibrotics in studies, (C) temporal comparison between studies conducted before and after 2014, and (D) comparison between IPF and non-IPF ILD.

The forest plot displays the individual study results, their respective weights, and the overall combined effect estimate represented by a diamond, with the confidence intervals for each study shown as horizontal lines.

Abbreviations: FVC, forced vital capacity; SD, standard deviation; CI, confidence interval; CARE-PF, Canadian Registry for Pulmonary Fibrosis; UCSF, ILD registry at the University of California, San Francisco; IPF, idiopathic pulmonary fibrosis; ILD, interstitial lung disease.

**Supplementary Figure 5** Forest Plot presenting the pooled mean difference for baseline DLCO.


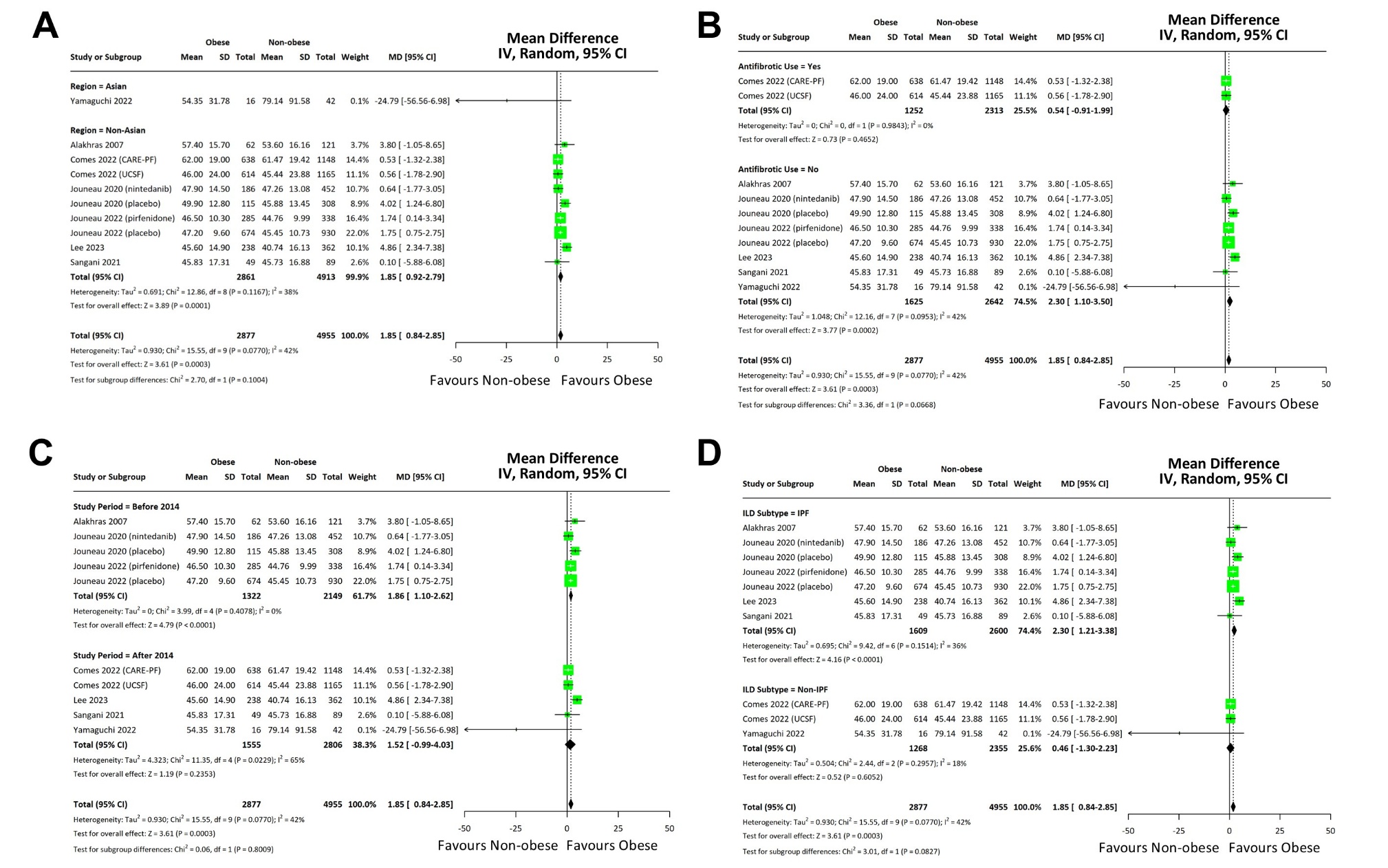


Obesity defined using region-specific cutoffs (Asia-Pacific ≥25; others ≥30 kg/m²).

DLCO was pooled as % predicted; studies reporting other units were converted or excluded.

Subgroups included (A) regional comparison between Asian and Non-Asian populations, (B) comparison based on the use of antifibrotics in studies, (C) temporal comparison between studies conducted before and after 2014, and (D) comparison between IPF and Non-IPF ILD.

The forest plot displays the individual study results, their respective weights, and the overall combined effect estimate represented by a diamond, with the confidence intervals for each study shown as horizontal lines.

Abbreviations: DLCO, diffusing capacity for carbon monoxide; SD, standard deviation; CI, confidence interval; CARE-PF, Canadian Registry for Pulmonary Fibrosis; UCSF, ILD registry at the University of California, San Francisco; IPF, idiopathic pulmonary fibrosis; ILD, interstitial lung disease.

**Supplementary Figure 6** Funnel Plot with trim and fill adjustment.

**
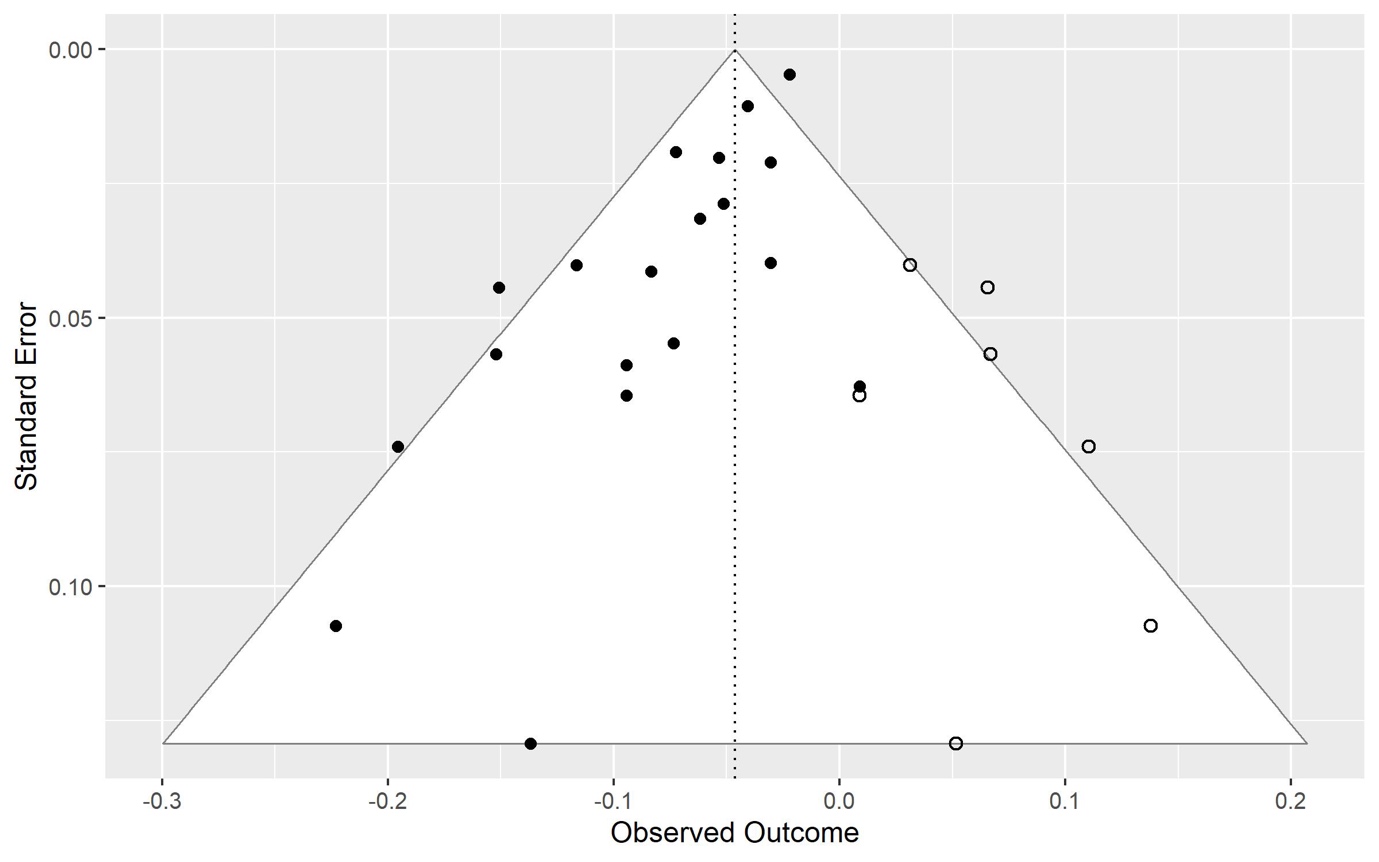
**

The vertical axis represents the standard error, and the horizontal axis shows the observed effect size. Black circles are observed studies, while white circles represent studies imputed using Duval and Tweedie's Trim and Fill method to adjust for suspected missing studies on the right. The plot illustrates the results after trim-and-fill adjustment for potential publication bias.

**Supplementary Table 1** Search strategy for PubMed/MEDLINE.

| **Search number** | **Query** | **Search details** | **Results** |
| --- | --- | --- | --- |
| 1 | Body Mass Index[MeSH Terms] | "body mass index"[MeSH Terms] | 149,717 |
| 2 | "quetelets index"[Title/Abstract] | "quetelets index"[Title/Abstract] | 4 |
| 3 | BMI[Title/Abstract] | "BMI"[Title/Abstract] | 197,178 |
| 4 | "body mass index"[Title/Abstract] | "body mass index"[Title/Abstract] | 240,684 |
| 5 | "quetelet index"[Title/Abstract] | "quetelet index"[Title/Abstract] | 497 |
| 6 | "quetelet s index"[Title/Abstract] | "quetelet s index"[Title/Abstract] | 223 |
| 7 | #2 OR #3 OR #4 OR #5 OR #6 | "quetelets index"[Title/Abstract] OR "BMI"[Title/Abstract] OR "body mass index"[Title/Abstract] OR "quetelet index"[Title/Abstract] OR "quetelet s index"[Title/Abstract] | 326,731 |
| 8 | #1 OR #7 | "body mass index"[MeSH Terms] OR "quetelets index"[Title/Abstract] OR "BMI"[Title/Abstract] OR "body mass index"[Title/Abstract] OR "quetelet index"[Title/Abstract] OR "quetelet s index"[Title/Abstract] | 368,131 |
| 9 | "body mass"[Title/Abstract] | "body mass"[Title/Abstract] | 278,257 |
| 10 | Weight Gain[MeSH Terms] | "weight gain"[MeSH Terms] | 36,853 |
| 11 | "weight gain*"[Title/Abstract] | "weight gain*"[Title/Abstract] | 78,558 |
| 12 | #10 OR #11 | "weight gain"[MeSH Terms] OR "weight gain*"[Title/Abstract] | 93,188 |
| 13 | Weight Loss[MeSH Terms] | "weight loss"[MeSH Terms] | 49,667 |
| 14 | "weight loss*"[Title/Abstract] | "weight loss*"[Title/Abstract] | 110,344 |
| 15 | "weight reduction*"[Title/Abstract] | "weight reduction*"[Title/Abstract] | 11,155 |
| 16 | #14 OR #15 | "weight loss*"[Title/Abstract] OR "weight reduction*"[Title/Abstract] | 117,432 |
| 17 | #13 OR #16 | "weight loss"[MeSH Terms] OR "weight loss*"[Title/Abstract] OR "weight reduction*"[Title/Abstract] | 133,285 |
| 18 | Body Weight[MeSH Terms] | "body weight"[MeSH Terms] | 534,463 |
| 19 | "body weight*"[Title/Abstract] | "body weight*"[Title/Abstract] | 250,500 |
| 20 | #18 OR #19 | "body weight"[MeSH Terms] OR "body weight*"[Title/Abstract] | 693,263 |
| 21 | Obesity[MeSH Terms] | "obesity"[MeSH Terms] | 262,791 |
| 22 | obes*[Title/Abstract] | "obes*"[Title/Abstract] | 391,189 |
| 23 | #21 OR #22 | "obesity"[MeSH Terms] OR "obes*"[Title/Abstract] | 444,160 |
| 24 | Overweight[MeSH Terms] | "overweight"[MeSH Terms] | 274,217 |
| 25 | overweight*[Title/Abstract] | "overweight*"[Title/Abstract] | 90,058 |
| 26 | "over weight*"[Title/Abstract] | "over weight*"[Title/Abstract] | 618 |
| 27 | #25 OR #26 | "overweight*"[Title/Abstract] OR "over weight*"[Title/Abstract] | 90,244 |
| 28 | #24 OR #27 | "overweight"[MeSH Terms] OR "overweight*"[Title/Abstract] OR "over weight*"[Title/Abstract] | 308,507 |
| 29 | fat[Title/Abstract] | "fat"[Title/Abstract] | 320,669 |
| 30 | #8 OR #9 OR #12 OR #17 OR #20 OR #23 OR #28 OR #29 | "body mass index"[MeSH Terms] OR "quetelets index"[Title/Abstract] OR "BMI"[Title/Abstract] OR "body mass index"[Title/Abstract] OR "quetelet index"[Title/Abstract] OR "quetelet s index"[Title/Abstract] OR "body mass"[Title/Abstract] OR "weight gain"[MeSH Terms] OR "weight gain*"[Title/Abstract] OR "weight loss"[MeSH Terms] OR "weight loss*"[Title/Abstract] OR "weight reduction*"[Title/Abstract] OR "body weight"[MeSH Terms] OR "body weight*"[Title/Abstract] OR "obesity"[MeSH Terms] OR "obes*"[Title/Abstract] OR "overweight"[MeSH Terms] OR "overweight*"[Title/Abstract] OR "over weight*"[Title/Abstract] OR "fat"[Title/Abstract] | 1,331,787 |
| 31 | Alveolitis, Extrinsic Allergic[MeSH Terms] | "alveolitis, extrinsic allergic"[MeSH Terms] | 4,642 |
| 32 | "extrinsic allergic alveoliti*"[Title/Abstract] | "extrinsic allergic alveoliti*"[Title/Abstract] | 627 |
| 33 | "hypersensitivity pneumoni*"[Title/Abstract] | "hypersensitivity pneumoni*"[Title/Abstract] | 3,098 |
| 34 | #32 OR #33 | "extrinsic allergic alveoliti*"[Title/Abstract] OR "hypersensitivity pneumoni*"[Title/Abstract] | 3,609 |
| 35 | #31 OR #34 | "alveolitis, extrinsic allergic"[MeSH Terms] OR "extrinsic allergic alveoliti*"[Title/Abstract] OR "hypersensitivity pneumoni*"[Title/Abstract] | 5,959 |
| 36 | "allergic pneumoniti*"[Title/Abstract] | "allergic pneumoniti*"[Title/Abstract] | 27 |
| 37 | Lung Diseases, Interstitial[MeSH Terms] | "lung diseases, interstitial"[MeSH Terms] | 85,389 |
| 38 | "interstitial lung disease*"[Title/Abstract] | "interstitial lung disease*"[Title/Abstract] | 16,434 |
| 39 | "interstitial pulmonary disease*"[Title/Abstract] | "interstitial pulmonary disease*"[Title/Abstract] | 314 |
| 40 | "interstitial pneumoni*"[Title/Abstract] | "interstitial pneumoni*"[Title/Abstract] | 11,965 |
| 41 | #38 OR #39 OR #40 | "interstitial lung disease*"[Title/Abstract] OR "interstitial pulmonary disease*"[Title/Abstract] OR "interstitial pneumoni*"[Title/Abstract] | 26,328 |
| 42 | #37 OR #41 | "lung diseases, interstitial"[MeSH Terms] OR "interstitial lung disease*"[Title/Abstract] OR "interstitial pulmonary disease*"[Title/Abstract] OR "interstitial pneumoni*"[Title/Abstract] | 96,240 |
| 43 | Idiopathic Interstitial Pneumonias[MeSH Terms] | "idiopathic interstitial pneumonias"[MeSH Terms] | 1,607 |
| 44 | "idiopathic interstitial pneumoni*"[Title/Abstract] | "idiopathic interstitial pneumoni*"[Title/Abstract] | 1,527 |
| 45 | #43 OR #44 | "idiopathic interstitial pneumonias"[MeSH Terms] OR "idiopathic interstitial pneumoni*"[Title/Abstract] | 2,751 |
| 46 | Hamman-Rich Syndrome[MeSH Terms] | "hamman rich syndrome"[MeSH Terms] | 14 |
| 47 | "hamman rich syndrome"[Title/Abstract] | "hamman rich syndrome"[Title/Abstract] | 230 |
| 48 | #46 OR #47 | "hamman rich syndrome"[MeSH Terms] OR "hamman rich syndrome"[Title/Abstract] | 242 |
| 49 | Idiopathic Pulmonary Fibrosis[MeSH Terms] | "idiopathic pulmonary fibrosis"[MeSH Terms] | 8,090 |
| 50 | "cryptogenic fibrosing alveoliti*"[Title/Abstract] | "cryptogenic fibrosing alveoliti*"[Title/Abstract] | 305 |
| 51 | "idiopathic fibrosing alveoliti*"[Title/Abstract] | "idiopathic fibrosing alveoliti*"[Title/Abstract] | 79 |
| 52 | "idiopathic pulmonary fibros*"[Title/Abstract] | "idiopathic pulmonary fibros*"[Title/Abstract] | 12,114 |
| 53 | #50 OR #51 OR #52 | "cryptogenic fibrosing alveoliti*"[Title/Abstract] OR "idiopathic fibrosing alveoliti*"[Title/Abstract] OR "idiopathic pulmonary fibros*"[Title/Abstract] | 12,450 |
| 54 | #49 OR #53 | "idiopathic pulmonary fibrosis"[MeSH Terms] OR "cryptogenic fibrosing alveoliti*"[Title/Abstract] OR "idiopathic fibrosing alveoliti*"[Title/Abstract] OR "idiopathic pulmonary fibros*"[Title/Abstract] | 14,613 |
| 55 | "fibrosing alveoliti*"[Title/Abstract] | "fibrosing alveoliti*"[Title/Abstract] | 901 |
| 56 | "idiopathic lung fibros*"[Title/Abstract] | "idiopathic lung fibros*"[Title/Abstract] | 79 |
| 57 | IPF[Title/Abstract] | "IPF"[Title/Abstract] | 8,807 |
| 58 | Pulmonary Fibrosis[MeSH Terms] | "pulmonary fibrosis"[MeSH Terms] | 28,316 |
| 59 | "pulmonary fibros*"[Title/Abstract] | "pulmonary fibros*"[Title/Abstract] | 25,373 |
| 60 | #58 OR #59 | "pulmonary fibrosis"[MeSH Terms] OR "pulmonary fibros*"[Title/Abstract] | 37,822 |
| 61 | "lung fibros*"[Title/Abstract] | "lung fibros*"[Title/Abstract] | 5,728 |
| 62 | "fibrocystic pulmonary dysplasia*"[Title/Abstract] | "fibrocystic pulmonary dysplasia*"[Title/Abstract] | 4 |
| 63 | "fibrotic lung disease*"[Title/Abstract] | "fibrotic lung disease*"[Title/Abstract] | 906 |
| 64 | "fibrotic pulmonary disease*"[Title/Abstract] | "fibrotic pulmonary disease*"[Title/Abstract] | 43 |
| 65 | "non specific interstitial pneumoni*"[Title/Abstract] | "non specific interstitial pneumoni*"[Title/Abstract] | 510 |
| 66 | IIP[Title/Abstract] | "IIP"[Title/Abstract] | 1,391 |
| 67 | ILD[Title/Abstract] | "ILD"[Title/Abstract] | 7,058 |
| 68 | IPFA[Title/Abstract] | "IPFA"[Title/Abstract] | 36 |
| 69 | NSIP[Title/Abstract] | "NSIP"[Title/Abstract] | 761 |
| 70 | UIP[Title/Abstract] | "UIP"[Title/Abstract] | 1,447 |
| 71 | #35 OR #36 OR #42 OR #45 OR #48 OR #54 OR #55 OR #56 OR #57 OR #60 OR #61 OR #62 OR #63 OR #64 OR #65 OR #66 OR #67 OR #68 OR #69 OR #70 | "alveolitis, extrinsic allergic"[MeSH Terms] OR "extrinsic allergic alveoliti*"[Title/Abstract] OR "hypersensitivity pneumoni*"[Title/Abstract] OR "allergic pneumoniti*"[Title/Abstract] OR "lung diseases, interstitial"[MeSH Terms] OR "interstitial lung disease*"[Title/Abstract] OR "interstitial pulmonary disease*"[Title/Abstract] OR "interstitial pneumoni*"[Title/Abstract] OR "idiopathic interstitial pneumonias"[MeSH Terms] OR "idiopathic interstitial pneumoni*"[Title/Abstract] OR "hamman rich syndrome"[MeSH Terms] OR "hamman rich syndrome"[Title/Abstract] OR "idiopathic pulmonary fibrosis"[MeSH Terms] OR "cryptogenic fibrosing alveoliti*"[Title/Abstract] OR "idiopathic fibrosing alveoliti*"[Title/Abstract] OR "idiopathic pulmonary fibros*"[Title/Abstract] OR "fibrosing alveoliti*"[Title/Abstract] OR "idiopathic lung fibros*"[Title/Abstract] OR "IPF"[Title/Abstract] OR "pulmonary fibrosis"[MeSH Terms] OR "pulmonary fibros*"[Title/Abstract] OR "lung fibros*"[Title/Abstract] OR "fibrocystic pulmonary dysplasia*"[Title/Abstract] OR "fibrotic lung disease*"[Title/Abstract] OR "fibrotic pulmonary disease*"[Title/Abstract] OR "non specific interstitial pneumoni*"[Title/Abstract] OR "IIP"[Title/Abstract] OR "ILD"[Title/Abstract] OR "IPFA"[Title/Abstract] OR "NSIP"[Title/Abstract] OR "UIP"[Title/Abstract] | 108,321 |
| 72 | #30 AND #71 | ("body mass index"[MeSH Terms] OR ("quetelets index"[Title/Abstract] OR "BMI"[Title/Abstract] OR "body mass index"[Title/Abstract] OR "quetelet index"[Title/Abstract] OR "quetelet s index"[Title/Abstract]) OR "body mass"[Title/Abstract] OR ("weight gain"[MeSH Terms] OR "weight gain*"[Title/Abstract]) OR ("weight loss"[MeSH Terms] OR ("weight loss*"[Title/Abstract] OR "weight reduction*"[Title/Abstract])) OR ("body weight"[MeSH Terms] OR "body weight*"[Title/Abstract]) OR ("obesity"[MeSH Terms] OR "obes*"[Title/Abstract]) OR ("overweight"[MeSH Terms] OR ("overweight*"[Title/Abstract] OR "over weight*"[Title/Abstract])) OR "fat"[Title/Abstract]) AND ("alveolitis, extrinsic allergic"[MeSH Terms] OR ("extrinsic allergic alveoliti*"[Title/Abstract] OR "hypersensitivity pneumoni*"[Title/Abstract]) OR "allergic pneumoniti*"[Title/Abstract] OR ("lung diseases, interstitial"[MeSH Terms] OR ("interstitial lung disease*"[Title/Abstract] OR "interstitial pulmonary disease*"[Title/Abstract] OR "interstitial pneumoni*"[Title/Abstract])) OR ("idiopathic interstitial pneumonias"[MeSH Terms] OR "idiopathic interstitial pneumoni*"[Title/Abstract]) OR ("hamman rich syndrome"[MeSH Terms] OR "hamman rich syndrome"[Title/Abstract]) OR ("idiopathic pulmonary fibrosis"[MeSH Terms] OR ("cryptogenic fibrosing alveoliti*"[Title/Abstract] OR "idiopathic fibrosing alveoliti*"[Title/Abstract] OR "idiopathic pulmonary fibros*"[Title/Abstract])) OR "fibrosing alveoliti*"[Title/Abstract] OR "idiopathic lung fibros*"[Title/Abstract] OR "IPF"[Title/Abstract] OR ("pulmonary fibrosis"[MeSH Terms] OR "pulmonary fibros*"[Title/Abstract]) OR "lung fibros*"[Title/Abstract] OR "fibrocystic pulmonary dysplasia*"[Title/Abstract] OR "fibrotic lung disease*"[Title/Abstract] OR "fibrotic pulmonary disease*"[Title/Abstract] OR "non specific interstitial pneumoni*"[Title/Abstract] OR "IIP"[Title/Abstract] OR "ILD"[Title/Abstract] OR "IPFA"[Title/Abstract] OR "NSIP"[Title/Abstract] OR "UIP"[Title/Abstract]) | 2,589 |
| 73 | #30 AND #71 | (("body mass index"[MeSH Terms] OR ("quetelets index"[Title/Abstract] OR "BMI"[Title/Abstract] OR "body mass index"[Title/Abstract] OR "quetelet index"[Title/Abstract] OR "quetelet s index"[Title/Abstract]) OR "body mass"[Title/Abstract] OR ("weight gain"[MeSH Terms] OR "weight gain*"[Title/Abstract]) OR ("weight loss"[MeSH Terms] OR ("weight loss*"[Title/Abstract] OR "weight reduction*"[Title/Abstract])) OR ("body weight"[MeSH Terms] OR "body weight*"[Title/Abstract]) OR ("obesity"[MeSH Terms] OR "obes*"[Title/Abstract]) OR ("overweight"[MeSH Terms] OR ("overweight*"[Title/Abstract] OR "over weight*"[Title/Abstract])) OR "fat"[Title/Abstract]) AND ("alveolitis, extrinsic allergic"[MeSH Terms] OR ("extrinsic allergic alveoliti*"[Title/Abstract] OR "hypersensitivity pneumoni*"[Title/Abstract]) OR "allergic pneumoniti*"[Title/Abstract] OR ("lung diseases, interstitial"[MeSH Terms] OR ("interstitial lung disease*"[Title/Abstract] OR "interstitial pulmonary disease*"[Title/Abstract] OR "interstitial pneumoni*"[Title/Abstract])) OR ("idiopathic interstitial pneumonias"[MeSH Terms] OR "idiopathic interstitial pneumoni*"[Title/Abstract]) OR ("hamman rich syndrome"[MeSH Terms] OR "hamman rich syndrome"[Title/Abstract]) OR ("idiopathic pulmonary fibrosis"[MeSH Terms] OR ("cryptogenic fibrosing alveoliti*"[Title/Abstract] OR "idiopathic fibrosing alveoliti*"[Title/Abstract] OR "idiopathic pulmonary fibros*"[Title/Abstract])) OR "fibrosing alveoliti*"[Title/Abstract] OR "idiopathic lung fibros*"[Title/Abstract] OR "IPF"[Title/Abstract] OR ("pulmonary fibrosis"[MeSH Terms] OR "pulmonary fibros*"[Title/Abstract]) OR "lung fibros*"[Title/Abstract] OR "fibrocystic pulmonary dysplasia*"[Title/Abstract] OR "fibrotic lung disease*"[Title/Abstract] OR "fibrotic pulmonary disease*"[Title/Abstract] OR "non specific interstitial pneumoni*"[Title/Abstract] OR "IIP"[Title/Abstract] OR "ILD"[Title/Abstract] OR "IPFA"[Title/Abstract] OR "NSIP"[Title/Abstract] OR "UIP"[Title/Abstract])) AND (alladult[Filter]) | 1,018 |

**Supplementary Table 2** Search strategy for Embase.

| **Search number** | **Query** | **Search details** | **Results** |
| --- | --- | --- | --- |
| 1 | 'quetelets index':ab,ti | 'quetelets index':ab,ti | 8 |
| 2 | 'bmi'/exp | 'bmi'/exp | 25 |
| 3 | bmi:ab,ti | bmi:ab,ti | 413,046 |
| 4 | #2 OR #3 | 'bmi'/exp OR bmi:ab,ti | 413,054 |
| 5 | 'body mass'/exp | 'body mass'/exp | 638,610 |
| 6 | 'body mass index':ab,ti | 'body mass index':ab,ti | 345,355 |
| 7 | 'quetelet index':ab,ti | 'quetelet index':ab,ti | 580 |
| 8 | 'quetelet s index':ab,ti | 'quetelet s index':ab,ti | 230 |
| 9 | 'body mass':ab,ti | 'body mass':ab,ti | 392,610 |
| 10 | #6 OR #7 OR #8 OR #9 | 'body mass index':ab,ti OR 'quetelet index':ab,ti OR 'quetelet s index':ab,ti OR 'body mass':ab,ti | 393,246 |
| 11 | #5 OR #10 | 'body mass'/exp OR ('body mass index':ab,ti OR 'quetelet index':ab,ti OR 'quetelet s index':ab,ti OR 'body mass':ab,ti) | 715,561 |
| 12 | 'body weight gain'/exp | 'body weight gain'/exp | 131,729 |
| 13 | 'weight gain*':ab,ti | 'weight gain*':ab,ti | 107,860 |
| 14 | #12 OR #13 | 'body weight gain'/exp OR 'weight gain*':ab,ti | 166,258 |
| 15 | 'body weight loss'/exp | 'body weight loss'/exp | 235,929 |
| 16 | 'weight loss*':ab,ti | 'weight loss*':ab,ti | 178,670 |
| 17 | 'weight reduction*':ab,ti | 'weight reduction*':ab,ti | 16,266 |
| 18 | #16 OR #17 | 'weight loss*':ab,ti OR 'weight reduction*':ab,ti | 188,970 |
| 19 | #15 OR #18 | 'body weight loss'/exp OR ('weight loss*':ab,ti OR 'weight reduction*':ab,ti) | 284,795 |
| 20 | 'body weight'/exp | 'body weight'/exp | 887,043 |
| 21 | 'body weight*':ab,ti | 'body weight*':ab,ti | 336,964 |
| 22 | #20 OR #21 | 'body weight'/exp OR 'body weight*':ab,ti | 1,014,487 |
| 23 | 'obesity'/exp | 'obesity'/exp | 676,662 |
| 24 | obes*:ab,ti | obes*:ab,ti | 566,270 |
| 25 | overweight*:ab,ti | overweight*:ab,ti | 133,702 |
| 26 | #24 OR #25 | obes*:ab,ti OR overweight*:ab,ti | 595,602 |
| 27 | #23 OR #26 | 'obesity'/exp OR (obes*:ab,ti OR overweight*:ab,ti) | 799,929 |
| 28 | 'over weight*':ab,ti | 'over weight*':ab,ti | 1,473 |
| 29 | fat:ab,ti | fat:ab,ti | 426,726 |
| 30 | #1 OR #4 OR #11 OR #14 OR #19 OR #22 OR #27 OR #28 OR #29 | 'quetelets index':ab,ti OR ('bmi'/exp OR bmi:ab,ti) OR ('body mass'/exp OR ('body mass index':ab,ti OR 'quetelet index':ab,ti OR 'quetelet s index':ab,ti OR 'body mass':ab,ti)) OR ('body weight gain'/exp OR 'weight gain*':ab,ti) OR ('body weight loss'/exp OR ('weight loss*':ab,ti OR 'weight reduction*':ab,ti)) OR ('body weight'/exp OR 'body weight*':ab,ti) OR ('obesity'/exp OR (obes*:ab,ti OR overweight*:ab,ti)) OR 'over weight*':ab,ti OR fat:ab,ti | 2,306,310 |
| 31 | 'allergic pneumonitis'/exp | 'allergic pneumonitis'/exp | 13,653 |
| 32 | 'extrinsic allergic alveoliti*':ab,ti | 'extrinsic allergic alveoliti*':ab,ti | 929 |
| 33 | 'hypersensitivity pneumoni*':ab,ti | 'hypersensitivity pneumoni*':ab,ti | 5,119 |
| 34 | 'allergic pneumoniti*':ab,ti | 'allergic pneumoniti*':ab,ti | 36 |
| 35 | #32 OR #33 OR #34 | 'extrinsic allergic alveoliti*':ab,ti OR 'hypersensitivity pneumoni*':ab,ti OR 'allergic pneumoniti*':ab,ti | 5,914 |
| 36 | #31 OR #35 | 'allergic pneumonitis'/exp OR ('extrinsic allergic alveoliti*':ab,ti OR 'hypersensitivity pneumoni*':ab,ti OR 'allergic pneumoniti*':ab,ti) | 14,613 |
| 37 | 'interstitial lung disease'/exp | 'interstitial lung disease'/exp | 141,846 |
| 38 | 'interstitial lung disease*':ab,ti | 'interstitial lung disease*':ab,ti | 28,605 |
| 39 | 'interstitial pulmonary disease*':ab,ti | 'interstitial pulmonary disease*':ab,ti | 457 |
| 40 | #38 OR #39 | 'interstitial lung disease*':ab,ti OR 'interstitial pulmonary disease*':ab,ti | 28,975 |
| 41 | #37 OR #40 | 'interstitial lung disease'/exp OR ('interstitial lung disease*':ab,ti OR 'interstitial pulmonary disease*':ab,ti) | 145,054 |
| 42 | 'interstitial pneumonia'/exp | 'interstitial pneumonia'/exp | 37,380 |
| 43 | 'interstitial pneumoni*':ab,ti | 'interstitial pneumoni*':ab,ti | 18,003 |
| 44 | 'idiopathic interstitial pneumoni*':ab,ti | 'idiopathic interstitial pneumoni*':ab,ti | 2,380 |
| 45 | #43 OR #44 | 'interstitial pneumoni*':ab,ti OR 'idiopathic interstitial pneumoni*':ab,ti | 18,003 |
| 46 | #42 OR #45 | 'interstitial pneumonia'/exp OR ('interstitial pneumoni*':ab,ti OR 'idiopathic interstitial pneumoni*':ab,ti) | 43,704 |
| 47 | 'fibrosing alveolitis'/exp | 'fibrosing alveolitis'/exp | 33,629 |
| 48 | 'hamman rich syndrome':ab,ti | 'hamman rich syndrome':ab,ti | 257 |
| 49 | 'cryptogenic fibrosing alveoliti*':ab,ti | 'cryptogenic fibrosing alveoliti*':ab,ti | 348 |
| 50 | 'idiopathic fibrosing alveoliti*':ab,ti | 'idiopathic fibrosing alveoliti*':ab,ti | 98 |
| 51 | 'idiopathic pulmonary fibros*':ab,ti | 'idiopathic pulmonary fibros*':ab,ti | 20,356 |
| 52 | 'fibrosing alveoliti*':ab,ti | 'fibrosing alveoliti*':ab,ti | 1,076 |
| 53 | 'idiopathic lung fibros*':ab,ti | 'idiopathic lung fibros*':ab,ti | 148 |
| 54 | ipf:ab,ti | ipf:ab,ti | 17,645 |
| 55 | #48 OR #49 OR #50 OR #51 OR #52 OR #53 OR #54 | 'hamman rich syndrome':ab,ti OR 'cryptogenic fibrosing alveoliti*':ab,ti OR 'idiopathic fibrosing alveoliti*':ab,ti OR 'idiopathic pulmonary fibros*':ab,ti OR 'fibrosing alveoliti*':ab,ti OR 'idiopathic lung fibros*':ab,ti OR ipf:ab,ti | 25,138 |
| 56 | #47 OR #55 | 'fibrosing alveolitis'/exp OR ('hamman rich syndrome':ab,ti OR 'cryptogenic fibrosing alveoliti*':ab,ti OR 'idiopathic fibrosing alveoliti*':ab,ti OR 'idiopathic pulmonary fibros*':ab,ti OR 'fibrosing alveoliti*':ab,ti OR 'idiopathic lung fibros*':ab,ti OR ipf:ab,ti) | 41,985 |
| 57 | 'lung fibrosis'/exp | 'lung fibrosis'/exp | 99,186 |
| 58 | 'pulmonary fibros*':ab,ti | 'pulmonary fibros*':ab,ti | 38,790 |
| 59 | 'lung fibros*':ab,ti | 'lung fibros*':ab,ti | 9,353 |
| 60 | #58 OR #59 | 'pulmonary fibros*':ab,ti OR 'lung fibros*':ab,ti | 43,771 |
| 61 | #57 OR #60 | 'lung fibrosis'/exp OR ('pulmonary fibros*':ab,ti OR 'lung fibros*':ab,ti) | 104,754 |
| 62 | 'fibrocystic pulmonary dysplasia*':ab,ti | 'fibrocystic pulmonary dysplasia*':ab,ti | 4 |
| 63 | 'fibrotic lung disease*':ab,ti | 'fibrotic lung disease*':ab,ti | 1,569 |
| 64 | 'fibrotic pulmonary disease*':ab,ti | 'fibrotic pulmonary disease*':ab,ti | 58 |
| 65 | 'non specific interstitial pneumoni*':ab,ti | 'non specific interstitial pneumoni*':ab,ti | 1,114 |
| 66 | iip:ab,ti | iip:ab,ti | 1,919 |
| 67 | ild:ab,ti | ild:ab,ti | 15,312 |
| 68 | ipfa:ab,ti | ipfa:ab,ti | 42 |
| 69 | nsip:ab,ti | nsip:ab,ti | 2,071 |
| 70 | uip:ab,ti | uip:ab,ti | 3,101 |
| 71 | #36 OR #41 OR #46 OR #56 OR #61 OR #62 OR #63 OR #64 OR #65 OR #66 OR #67 OR #68 OR #69 OR #70 | ('allergic pneumonitis'/exp OR ('extrinsic allergic alveoliti*':ab,ti OR 'hypersensitivity pneumoni*':ab,ti OR 'allergic pneumoniti*':ab,ti)) OR ('interstitial lung disease'/exp OR ('interstitial lung disease*':ab,ti OR 'interstitial pulmonary disease*':ab,ti)) OR ('interstitial pneumonia'/exp OR ('interstitial pneumoni*':ab,ti OR 'idiopathic interstitial pneumoni*':ab,ti)) OR ('fibrosing alveolitis'/exp OR ('hamman rich syndrome':ab,ti OR 'cryptogenic fibrosing alveoliti*':ab,ti OR 'idiopathic fibrosing alveoliti*':ab,ti OR 'idiopathic pulmonary fibros*':ab,ti OR 'fibrosing alveoliti*':ab,ti OR 'idiopathic lung fibros*':ab,ti OR ipf:ab,ti)) OR ('lung fibrosis'/exp OR ('pulmonary fibros*':ab,ti OR 'lung fibros*':ab,ti)) OR 'fibrocystic pulmonary dysplasia*':ab,ti OR 'fibrotic lung disease*':ab,ti OR 'fibrotic pulmonary disease*':ab,ti OR 'non specific interstitial pneumoni*':ab,ti OR iip:ab,ti OR ild:ab,ti OR ipfa:ab,ti OR nsip:ab,ti OR uip:ab,ti | 211,733 |
| 72 | #30 AND #71 | ('quetelets index':ab,ti OR ('bmi'/exp OR bmi:ab,ti) OR ('body mass'/exp OR ('body mass index':ab,ti OR 'quetelet index':ab,ti OR 'quetelet s index':ab,ti OR 'body mass':ab,ti)) OR ('body weight gain'/exp OR 'weight gain*':ab,ti) OR ('body weight loss'/exp OR ('weight loss*':ab,ti OR 'weight reduction*':ab,ti)) OR ('body weight'/exp OR 'body weight*':ab,ti) OR ('obesity'/exp OR (obes*:ab,ti OR overweight*:ab,ti)) OR 'over weight*':ab,ti OR fat:ab,ti) AND (('allergic pneumonitis'/exp OR ('extrinsic allergic alveoliti*':ab,ti OR 'hypersensitivity pneumoni*':ab,ti OR 'allergic pneumoniti*':ab,ti)) OR ('interstitial lung disease'/exp OR ('interstitial lung disease*':ab,ti OR 'interstitial pulmonary disease*':ab,ti)) OR ('interstitial pneumonia'/exp OR ('interstitial pneumoni*':ab,ti OR 'idiopathic interstitial pneumoni*':ab,ti)) OR ('fibrosing alveolitis'/exp OR ('hamman rich syndrome':ab,ti OR 'cryptogenic fibrosing alveoliti*':ab,ti OR 'idiopathic fibrosing alveoliti*':ab,ti OR 'idiopathic pulmonary fibros*':ab,ti OR 'fibrosing alveoliti*':ab,ti OR 'idiopathic lung fibros*':ab,ti OR ipf:ab,ti)) OR ('lung fibrosis'/exp OR ('pulmonary fibros*':ab,ti OR 'lung fibros*':ab,ti)) OR 'fibrocystic pulmonary dysplasia*':ab,ti OR 'fibrotic lung disease*':ab,ti OR 'fibrotic pulmonary disease*':ab,ti OR 'non specific interstitial pneumoni*':ab,ti OR iip:ab,ti OR ild:ab,ti OR ipfa:ab,ti OR nsip:ab,ti OR uip:ab,ti) | 13,248 |
| 73 | #30 AND #71 AND ([adult]/lim OR [young adult]/lim OR [middle aged]/lim OR [aged]/lim OR [very elderly]/lim) | ('quetelets index':ab,ti OR ('bmi'/exp OR bmi:ab,ti) OR ('body mass'/exp OR ('body mass index':ab,ti OR 'quetelet index':ab,ti OR 'quetelet s index':ab,ti OR 'body mass':ab,ti)) OR ('body weight gain'/exp OR 'weight gain*':ab,ti) OR ('body weight loss'/exp OR ('weight loss*':ab,ti OR 'weight reduction*':ab,ti)) OR ('body weight'/exp OR 'body weight*':ab,ti) OR ('obesity'/exp OR (obes*:ab,ti OR overweight*:ab,ti)) OR 'over weight*':ab,ti OR fat:ab,ti) AND (('allergic pneumonitis'/exp OR ('extrinsic allergic alveoliti*':ab,ti OR 'hypersensitivity pneumoni*':ab,ti OR 'allergic pneumoniti*':ab,ti)) OR ('interstitial lung disease'/exp OR ('interstitial lung disease*':ab,ti OR 'interstitial pulmonary disease*':ab,ti)) OR ('interstitial pneumonia'/exp OR ('interstitial pneumoni*':ab,ti OR 'idiopathic interstitial pneumoni*':ab,ti)) OR ('fibrosing alveolitis'/exp OR ('hamman rich syndrome':ab,ti OR 'cryptogenic fibrosing alveoliti*':ab,ti OR 'idiopathic fibrosing alveoliti*':ab,ti OR 'idiopathic pulmonary fibros*':ab,ti OR 'fibrosing alveoliti*':ab,ti OR 'idiopathic lung fibros*':ab,ti OR ipf:ab,ti)) OR ('lung fibrosis'/exp OR ('pulmonary fibros*':ab,ti OR 'lung fibros*':ab,ti)) OR 'fibrocystic pulmonary dysplasia*':ab,ti OR 'fibrotic lung disease*':ab,ti OR 'fibrotic pulmonary disease*':ab,ti OR 'non specific interstitial pneumoni*':ab,ti OR iip:ab,ti OR ild:ab,ti OR ipfa:ab,ti OR nsip:ab,ti OR uip:ab,ti) AND ([adult]/lim OR [young adult]/lim OR [middle aged]/lim OR [aged]/lim OR [very elderly]/lim) | 7,198 |

**Supplementary Table 3** Search strategy for the Cochrane Library.

| **Search number** | **Query** | **Search details** | **Results** |
| --- | --- | --- | --- |
| 1 | MeSH descriptor: [Body Mass Index] explode all trees | [mh "Body Mass Index"] | 12,436 |
| 2 | quetelets index:ab,ti | "quetelets index":ab,ti | 5 |
| 3 | BMI:ab,ti | BMI:ab,ti | 55,431 |
| 4 | body mass index:ab,ti | "body mass index":ab,ti | 41,087 |
| 5 | quetelet index:ab,ti | "quetelet index":ab,ti | 56 |
| 6 | quetelet s index:ab,ti | "quetelet s index":ab,ti | 5 |
| 7 | #2 OR #3 OR #4 OR #5 OR #6 | ("quetelets index":ab,ti OR BMI:ab,ti OR "body mass index":ab,ti OR "quetelet index":ab,ti OR "quetelet s index":ab,ti) | 77,800 |
| 8 | #1 OR #7 | ([mh "Body Mass Index"] OR ("quetelets index":ab,ti OR BMI:ab,ti OR "body mass index":ab,ti OR "quetelet index":ab,ti OR "quetelet s index":ab,ti)) | 82,066 |
| 9 | body mass:ab,ti | "body mass":ab,ti | 48,111 |
| 10 | MeSH descriptor: [Weight Gain] explode all trees | [mh "Weight Gain"] | 3,312 |
| 11 | (weight next gain*):ab,ti | (weight next gain*):ab,ti | 11,476 |
| 12 | #10 OR #11 | ([mh "Weight Gain"] OR (weight next gain*):ab,ti) | 12,378 |
| 13 | MeSH descriptor: [Weight Loss] explode all trees | [mh "Weight Loss"] | 8,188 |
| 14 | (weight next loss*):ab,ti | (weight next loss*):ab,ti | 21,839 |
| 15 | (weight next reduction*):ab,ti | (weight next reduction*):ab,ti | 3,079 |
| 16 | #14 OR #15 | ((weight next loss*):ab,ti OR (weight next reduction*):ab,ti) | 23,438 |
| 17 | #13 OR #16 | ([mh "Weight Loss"] OR ((weight next loss*):ab,ti OR (weight next reduction*):ab,ti)) | 24,912 |
| 18 | MeSH descriptor: [Body Weight] explode all trees | [mh "Body Weight"] | 41,393 |
| 19 | (body next weight*):ab,ti | (body next weight*):ab,ti | 37,292 |
| 20 | #18 OR #19 | ([mh "Body Weight"] OR (body next weight*):ab,ti) | 69,182 |
| 21 | MeSH descriptor: [Obesity] explode all trees | [mh Obesity] | 21,415 |
| 22 | obes*:ab,ti | obes*:ab,ti | 47,103 |
| 23 | #21 OR #22 | [mh Obesity] OR obes*:ab,ti | 50,714 |
| 24 | MeSH descriptor: [Overweight] explode all trees | [mh Overweight] | 24,827 |
| 25 | overweight*:ab,ti | overweight*:ab,ti | 19,960 |
| 26 | (over next weight*):ab,ti | (over next weight*):ab,ti | 189 |
| 27 | #25 OR #26 | (overweight*:ab,ti OR (over next weight*):ab,ti) | 20,029 |
| 28 | #24 OR #27 | ([mh Overweight] OR (overweight*:ab,ti OR (over next weight*):ab,ti)) | 35,149 |
| 29 | fat:ab,ti | fat:ab,ti | 38,790 |
| 30 | #8 OR #9 OR #12 OR #17 OR #20 OR #23 OR #28 OR #29 | (([mh "Body Mass Index"] OR ("quetelets index":ab,ti OR BMI:ab,ti OR "body mass index":ab,ti OR "quetelet index":ab,ti OR "quetelet s index":ab,ti)) OR "body mass":ab,ti OR ([mh "Weight Gain"] OR (weight next gain*):ab,ti) OR ([mh "Weight Loss"] OR ((weight next loss*):ab,ti OR (weight next reduction*):ab,ti)) OR ([mh "Body Weight"] OR (body next weight*):ab,ti) OR [mh Obesity] OR obes*:ab,ti OR ([mh Overweight] OR (overweight*:ab,ti OR (over next weight*):ab,ti)) OR fat:ab,ti) | 172,200 |
| 31 | MeSH descriptor: [Alveolitis, Extrinsic Allergic] explode all trees | [mh "Alveolitis, Extrinsic Allergic"] | 30 |
| 32 | (extrinsic next allergic next alveoliti*):ab,ti | (extrinsic next allergic next alveoliti*):ab,ti | 1 |
| 33 | (hypersensitivity next pneumoni*):ab,ti | (hypersensitivity next pneumoni*):ab,ti | 64 |
| 34 | #32 OR #33 | ((extrinsic next allergic next alveoliti*):ab,ti OR (hypersensitivity next pneumoni*):ab,ti) | 65 |
| 35 | #31 OR #34 | ([mh "Alveolitis, Extrinsic Allergic"] OR ((extrinsic next allergic next alveoliti*):ab,ti OR (hypersensitivity next pneumoni*):ab,ti)) | 82 |
| 36 | (allergic next pneumoniti*):ab,ti | (allergic next pneumoniti*):ab,ti | - |
| 37 | MeSH descriptor: [Lung Diseases, Interstitial] explode all trees | [mh "Lung Diseases, Interstitial"] | 1,573 |
| 38 | (interstitial next lung next disease*):ab,ti | (interstitial next lung next disease*):ab,ti | 1,381 |
| 39 | (interstitial next pulmonary next disease*):ab,ti | (interstitial next pulmonary next disease*):ab,ti | 46 |
| 40 | (interstitial next pneumoni*):ab,ti | (interstitial next pneumoni*):ab,ti | 463 |
| 41 | #38 OR #39 OR #40 | ((interstitial next lung next disease*):ab,ti OR (interstitial next pulmonary next disease*):ab,ti OR (interstitial next pneumoni*):ab,ti) | 1,777 |
| 42 | #37 OR #41 | ([mh "Lung Diseases, Interstitial"] OR ((interstitial next lung next disease*):ab,ti OR (interstitial next pulmonary next disease*):ab,ti OR (interstitial next pneumoni*):ab,ti)) | 2,920 |
| 43 | MeSH descriptor: [Idiopathic Interstitial Pneumonias] explode all trees | [mh "Idiopathic Interstitial Pneumonias"] | 181 |
| 44 | (idiopathic next interstitial next pneumoni*):ab,ti | (idiopathic next interstitial next pneumoni*):ab,ti | 72 |
| 45 | #43 OR #44 | ([mh "Idiopathic Interstitial Pneumonias"] OR (idiopathic next interstitial next pneumoni*):ab,ti) | 242 |
| 46 | MeSH descriptor: [Hamman-Rich Syndrome] explode all trees | [mh "Hamman-Rich Syndrome"] | - |
| 47 | hamman rich syndrome:ab,ti | "hamman rich syndrome":ab,ti | - |
| 48 | #46 OR #47 | ([mh "Hamman-Rich Syndrome"] OR "hamman rich syndrome":ab,ti) | - |
| 49 | MeSH descriptor: [Idiopathic Pulmonary Fibrosis] explode all trees | [mh "Idiopathic Pulmonary Fibrosis"] | 499 |
| 50 | (cryptogenic next fibrosing next alveoliti*):ab,ti | (cryptogenic next fibrosing next alveoliti*):ab,ti | 10 |
| 51 | (idiopathic next fibrosing next alveoliti*):ab,ti | (idiopathic next fibrosing next alveoliti*):ab,ti | 1 |
| 52 | (idiopathic next pulmonary next fibros*):ab,ti | (idiopathic next pulmonary next fibros*):ab,ti | 1,418 |
| 53 | #50 OR #51 OR #52 | ((cryptogenic next fibrosing next alveoliti*):ab,ti OR (idiopathic next fibrosing next alveoliti*):ab,ti OR (idiopathic next pulmonary next fibros*):ab,ti) | 1,429 |
| 54 | #49 OR #53 | ([mh "Idiopathic Pulmonary Fibrosis"] OR ((cryptogenic next fibrosing next alveoliti*):ab,ti OR (idiopathic next fibrosing next alveoliti*):ab,ti OR (idiopathic next pulmonary next fibros*):ab,ti)) | 1,503 |
| 55 | (fibrosing next alveoliti*):ab,ti | (fibrosing next alveoliti*):ab,ti | 19 |
| 56 | (idiopathic next lung next fibros*):ab,ti | (idiopathic next lung next fibros*):ab,ti | 5 |
| 57 | IPF:ab,ti | IPF:ab,ti | 1,300 |
| 58 | MeSH descriptor: [Pulmonary Fibrosis] explode all trees | [mh "Pulmonary Fibrosis"] | 782 |
| 59 | (pulmonary next fibros*):ab,ti | (pulmonary next fibros*):ab,ti | 1,785 |
| 60 | #58 OR #59 | ([mh "Pulmonary Fibrosis"] OR (pulmonary next fibros*):ab,ti) | 2,006 |
| 61 | (lung next fibros*):ab,ti | (lung next fibros*):ab,ti | 217 |
| 62 | (fibrocystic next pulmonary next dysplasia*):ab,ti | (fibrocystic next pulmonary next dysplasia*):ab,ti | - |
| 63 | (fibrotic next lung next disease*):ab,ti | (fibrotic next lung next disease*):ab,ti | 52 |
| 64 | (fibrotic next pulmonary next disease*):ab,ti | (fibrotic next pulmonary next disease*):ab,ti | 2 |
| 65 | (non next specific next interstitial next pneumoni*):ab,ti | (non next specific next interstitial next pneumoni*):ab,ti | 28 |
| 66 | IIP:ab,ti | IIP:ab,ti | 184 |
| 67 | ILD:ab,ti | ILD:ab,ti | 891 |
| 68 | IPFA:ab,ti | IPFA:ab,ti | 11 |
| 69 | NSIP:ab,ti | NSIP:ab,ti | 45 |
| 70 | UIP:ab,ti | UIP:ab,ti | 130 |
| 71 | #35 OR #36 OR #42 OR #45 OR #48 OR #54 OR #55 OR #56 OR #57 OR #60 OR #61 OR #62 OR #63 OR #64 OR #65 OR #66 OR #67 OR #68 OR #69 OR #70 | (([mh "Alveolitis, Extrinsic Allergic"] OR ((extrinsic next allergic next alveoliti*):ab,ti OR (hypersensitivity next pneumoni*):ab,ti)) OR (allergic next pneumoniti*):ab,ti OR ([mh "Lung Diseases, Interstitial"] OR ((interstitial next lung next disease*):ab,ti OR (interstitial next pulmonary next disease*):ab,ti OR (interstitial next pneumoni*):ab,ti)) OR ([mh "Idiopathic Interstitial Pneumonias"] OR (idiopathic next interstitial next pneumoni*):ab,ti) OR ([mh "Hamman-Rich Syndrome"] OR "hamman rich syndrome":ab,ti) OR ([mh "Idiopathic Pulmonary Fibrosis"] OR ((cryptogenic next fibrosing next alveoliti*):ab,ti OR (idiopathic next fibrosing next alveoliti*):ab,ti OR (idiopathic next pulmonary next fibros*):ab,ti)) OR (fibrosing next alveoliti*):ab,ti OR (idiopathic next lung next fibros*):ab,ti OR IPF:ab,ti OR ([mh "Pulmonary Fibrosis"] OR (pulmonary next fibros*):ab,ti) OR (lung next fibros*):ab,ti OR (fibrocystic next pulmonary next dysplasia*):ab,ti OR (fibrotic next lung next disease*):ab,ti OR (fibrotic next pulmonary next disease*):ab,ti OR (non next specific next interstitial next pneumoni*):ab,ti OR IIP:ab,ti OR ILD:ab,ti OR IPFA:ab,ti OR NSIP:ab,ti OR UIP:ab,ti) | 4,395 |
| 72 | #30 AND #71 | (((([mh "Body Mass Index"] OR ("quetelets index":ab,ti OR BMI:ab,ti OR "body mass index":ab,ti OR "quetelet index":ab,ti OR "quetelet s index":ab,ti)) OR "body mass":ab,ti OR ([mh "Weight Gain"] OR (weight next gain*):ab,ti) OR ([mh "Weight Loss"] OR ((weight next loss*):ab,ti OR (weight next reduction*):ab,ti)) OR ([mh "Body Weight"] OR (body next weight*):ab,ti) OR [mh Obesity] OR obes*:ab,ti OR ([mh Overweight] OR (overweight*:ab,ti OR (over next weight*):ab,ti)) OR fat:ab,ti)) AND ((([mh "Alveolitis, Extrinsic Allergic"] OR ((extrinsic next allergic next alveoliti*):ab,ti OR (hypersensitivity next pneumoni*):ab,ti)) OR (allergic next pneumoniti*):ab,ti OR ([mh "Lung Diseases, Interstitial"] OR ((interstitial next lung next disease*):ab,ti OR (interstitial next pulmonary next disease*):ab,ti OR (interstitial next pneumoni*):ab,ti)) OR ([mh "Idiopathic Interstitial Pneumonias"] OR (idiopathic next interstitial next pneumoni*):ab,ti) OR ([mh "Hamman-Rich Syndrome"] OR "hamman rich syndrome":ab,ti) OR ([mh "Idiopathic Pulmonary Fibrosis"] OR ((cryptogenic next fibrosing next alveoliti*):ab,ti OR (idiopathic next fibrosing next alveoliti*):ab,ti OR (idiopathic next pulmonary next fibros*):ab,ti)) OR (fibrosing next alveoliti*):ab,ti OR (idiopathic next lung next fibros*):ab,ti OR IPF:ab,ti OR ([mh "Pulmonary Fibrosis"] OR (pulmonary next fibros*):ab,ti) OR (lung next fibros*):ab,ti OR (fibrocystic next pulmonary next dysplasia*):ab,ti OR (fibrotic next lung next disease*):ab,ti OR (fibrotic next pulmonary next disease*):ab,ti OR (non next specific next interstitial next pneumoni*):ab,ti OR IIP:ab,ti OR ILD:ab,ti OR IPFA:ab,ti OR NSIP:ab,ti OR UIP:ab,ti))) | 213 |
| 73 | MeSH descriptor: [Adult] explode all trees | [mh Adult] | 586,627 |
| 74 | #72 AND #73 | ((((([mh "Body Mass Index"] OR ("quetelets index":ab,ti OR BMI:ab,ti OR "body mass index":ab,ti OR "quetelet index":ab,ti OR "quetelet s index":ab,ti)) OR "body mass":ab,ti OR ([mh "Weight Gain"] OR (weight next gain*):ab,ti) OR ([mh "Weight Loss"] OR ((weight next loss*):ab,ti OR (weight next reduction*):ab,ti)) OR ([mh "Body Weight"] OR (body next weight*):ab,ti) OR [mh Obesity] OR obes*:ab,ti OR ([mh Overweight] OR (overweight*:ab,ti OR (over next weight*):ab,ti)) OR fat:ab,ti)) AND ((([mh "Alveolitis, Extrinsic Allergic"] OR ((extrinsic next allergic next alveoliti*):ab,ti OR (hypersensitivity next pneumoni*):ab,ti)) OR (allergic next pneumoniti*):ab,ti OR ([mh "Lung Diseases, Interstitial"] OR ((interstitial next lung next disease*):ab,ti OR (interstitial next pulmonary next disease*):ab,ti OR (interstitial next pneumoni*):ab,ti)) OR ([mh "Idiopathic Interstitial Pneumonias"] OR (idiopathic next interstitial next pneumoni*):ab,ti) OR ([mh "Hamman-Rich Syndrome"] OR "hamman rich syndrome":ab,ti) OR ([mh "Idiopathic Pulmonary Fibrosis"] OR ((cryptogenic next fibrosing next alveoliti*):ab,ti OR (idiopathic next fibrosing next alveoliti*):ab,ti OR (idiopathic next pulmonary next fibros*):ab,ti)) OR (fibrosing next alveoliti*):ab,ti OR (idiopathic next lung next fibros*):ab,ti OR IPF:ab,ti OR ([mh "Pulmonary Fibrosis"] OR (pulmonary next fibros*):ab,ti) OR (lung next fibros*):ab,ti OR (fibrocystic next pulmonary next dysplasia*):ab,ti OR (fibrotic next lung next disease*):ab,ti OR (fibrotic next pulmonary next disease*):ab,ti OR (non next specific next interstitial next pneumoni*):ab,ti OR IIP:ab,ti OR ILD:ab,ti OR IPFA:ab,ti OR NSIP:ab,ti OR UIP:ab,ti))) AND [mh Adult]) | 57 |

**Supplementary Table 4** Baseline characteristics by obesity status of the included studies.

| Studies | | | Obese | | | | | Non-obese | | | | | Definition of Obesity (BMI ≥, kg/m^2^) |
| --- | --- | --- | --- | --- | --- | --- | --- | --- | --- | --- | --- | --- | --- |
|  |  |  | Age | Number | Male (%) | Baseline FVC, % predicted | Baseline DLCO, % predicted | Age | Number | Male (%) | Baseline FVC, % predicted | Baseline DLCO, % predicted |  |
| Alakhras, 2007 | | | 69.3 ± 8.5 | 66 | 68.2 | 74.6 ± 19.1 | 57.4 ± 15.7 | 72.4 ± 8.9 | 131 | 69.5 | 72.5 ± 19.1 | 53.6 ± 16.2 | 30 |
| Comes, 2022 | CARE-PF | | 62.0 ± 11.0 | 638 | 48.4 | 77.0 ± 18.0 | 62.0 ± 19.0 | 61.3 ± 12.8 | 1148 | 44.7 | 79.3 ± 20.0 | 61.5 ± 19.4 | 30 |
|  | UCSF | | 63.0 ± 11.0 | 614 | 53.6 | 68.0 ± 22.0 | 46.0 ± 24.0 | 66.7 ± 1.9 | 1165 | 57.1 | 71.2 ± 14.5 | 45.4 ± 23.8 | 30 |
| Jouneau, 2020 | | nintedanib | 65.2 ± 7.5 | 186 | 76.3 | 75.0 ± 16.5 | 47.9 ± 14.5 | 67.2 ± 8.3 | 452 | 80.8 | 81.6 ± 17.7 | 47.3 ± 13.0 | 30 |
|  |  | placebo | 66.4 ± 7.6 | 115 | 82.6 | 78.7 ± 18.6 | 49.9 ± 12.8 | 67.2 ± 8.0 | 308 | 77.6 | 79.2 ± 18.2 | 45.9 ± 13.4 | 30 |
| Jouneau, 2022 | | placebo | 65.2 ± 7.6 | 674 | 71.8 | 72.1 ± 13.0 | 47.2 ± 9.6 | 68.0 ± 7.0 | 930 | 74.6 | 72.4 ± 13.5 | 45.5 ± 10.7 | 30 |
|  |  | pirfenidone | 65.6 ± 7.7 | 285 | 72.3 | 70.7 ± 11.8 | 46.5 ± 10.3 | 69.3 ± 7.2 | 338 | 76.0 | 72.3 ± 14.3 | 44.8 ± 10.0 | 30 |
| Lee, 2023 | | | 69.2 ± 7.7 | 238 | 70.6 | 70 ± 16.6 | 45.6 ± 14.9 | 72.2 ± 7.7 | 362 | 76.5 | 70.5 ± 16.7 | 40.7 ± 15.9 | 30 |
| Sangani, 2021 | | | 73.27 ± 9.12 | 49 | 65.3 | 75.24 ± 15.6 | 45.83 ± 17.31 | 77.97 ± 9.59 | 89 | 57.3 | 70.4 ± 19.8 | 45.7 ± 16.9 | 30 |
| Yamaguchi, 2022 | | | 51 ± 10 | 16 | 25 | 72.4 (53.6 – 97.1) | 52.5 (35.5 – 74.6) | 56 ± 15 | 42 | 26.2 | 81.7 (42.0 – 121.0) | 83.5 (17.7 – 137) | 25 |
| Yoon, 2024 | | | 68.0 ± 7.7 | 4307 | 73.5 | NA | NA | 69.4 ± 8.3 | 7519 | 74.1 | NA | NA | 25 |

Notes: Data are presented as mean ± standard deviation, median (interquartile range), or number (%). Where mean and standard deviation were unavailable, median values were reported as provided in the original studies.

Abbreviations: BMI, body mass index; FVC, forced vital capacity; DLCO, diffusing capacity for carbon monoxide; CARE-PF, the Canadian Registry for Pulmonary Fibrosis; UCSF, the ILD registry at the University of California, San Francisco; NA, not available.

**Supplementary Table 5** Quality assessment of observational studies using the nine-star Newcastle-Ottawa Scale.

| Study | Selection | | | | Comparability | | Outcome | | | Total Stars |
| --- | --- | --- | --- | --- | --- | --- | --- | --- | --- | --- |
|  | Representativeness of the exposed cohort | Selection of the non-exposed cohort | Ascertainment of exposure | Outcome of interest not present at start of study | Comparability of cohorts on the basis of the design or analysis | Comparability (adjustment for additional variables) | Assessment of outcome | Long enough follow-up | Adequacy of follow-up |  |
| Alakhras, 2007 |  | * | * | * | * | * | * |  |  | 6 |
| Alhamad, 2020 | * | * | * | * |  | * | * |  |  | 6 |
| Aono, 2020 | * | * | * | * |  | * | * | * | * | 8 |
| Comes, 2022 |  | * | * | * |  | * | * | * | * | 7 |
| Gao, 2021 | * | * | * | * |  | * | * | * |  | 7 |
| Ikezoe, 2017 |  | * | * | * |  |  | * | * | * | 6 |
| Jalaber, 2021 | * | * | * |  |  |  | * |  |  | 4 |
| Jouneau, 2020 | * | * | * | * | * | * | * | * | * | 9 |
| Jouneau, 2022 |  | * | * | * |  | * | * | * |  | 6 |
| Jouneau, 2022 (2) | * | * | * |  |  | * | * | * |  | 6 |
| Kim, 2021 | * | * | * | * |  |  | * |  |  | 5 |
| Kishiba, 2021 |  | * | * | * |  |  | * | * |  | 5 |
| Kono, 2023 | * | * | * |  |  | * | * | * |  | 6 |
| Lee, 2023 | * | * | * | * |  | * | * | * |  | 7 |
| Li, 2019 | * | * | * | * |  | * | * |  | * | 7 |
| Sangani, 2021 | * | * | * | * |  | * | * | * |  | 7 |
| Suzuki, 2018 | * | * | * |  |  | * | * | * |  | 6 |
| Suzuki, 2021 | * | * | * | * |  | * | * |  | * | 7 |
| Suzuki, 2021 (2) | * | * | * |  |  | * | * |  |  | 5 |
| Snyder, 2019 | * | * | * | * |  | * | * | * |  | 7 |
| Yamaguchi, 2022 | * | * | * |  | * |  | * | * |  | 6 |
| Yamazaki, 2022 | * | * | * | * |  |  | * | * | * | 7 |
| Yoon, 2024 |  | * | * | * |  | * | * | * | * | 7 |
| Zinellu, 2021 | * | * | * |  |  | * | * | * |  | 6 |
| Zinellu, 2022 | * | * | * |  |  | * | * | * |  | 6 |

Stars were summed according to a modified Newcastle-Ottawa Scale. A maximum of one star was awarded for comparability.

**Supplementary Table 6** Exploratory assessment of publication bias for baseline FVC and DLCO

|  | Baseline FVC | Baseline DLCO |
| --- | --- | --- |
| Egger’s test z -value | 0.698 | -0.744 |
| p-value for Egger’s test | 0.485 | 0.457 |
| Number of imputed studies | 0 | 1 |
| Adjusted pooled MD (95% CI) | -1.58 (-3.19 to 0.03) | 1.90 (0.99 to 2.81) |
| Adjusted p-value (pooed effect) | 0.055 | <0.001 |

Abbreviations: FVC, forced vital capacity; DLCO, diffusing capacity for carbon monoxide; MD, mean difference; CI, confidence interval.

**Supplementary Table 7** Sensitivity analysis comparing pooled estimates using DerSimonian-Laird (DL) and restricted maximum likelihood (REML) estimators.

| Outcome | DL method | | REML method | | | |
| --- | --- | --- | --- | --- | --- | --- |
|  | Effect (95% CI) | p-value | | Effect (95% CI) | p-value |  |
| Mortality (RR) | 0.91 (0.87 to 0.94) | <0.001 | | 0.91 (0.87 to 0.94) | <0.001 |  |
| Mortality (HR, BMI as continuous variable) | 0.94 (0.92 to 0.96) | <0.001 | | 0.94 (0.92 to 0.96) | <0.001 |  |
| Mortality (HR, obese vs. non-obese) | 0.75 (0.55 to 1.02) | 0.696 | | 0.75 (0.56 to 1.02) | 0.066 |  |
| Hospitalization (HR, univariable) | 0.97 (0.95 to 0.99) | 0.013 | | 0.97 (0.93 to 1.00) | 0.043 |  |
| Hospitalization (HR, multivariable) | 0.98 (0.95 to 1.00) | 0.075 | | 0.96 (0.91 to 1.02) | 0.192 |  |
| Baseline FVC (MD, % predicted) | -1.61 (-3.10 to -0.12) | 0.034 | | -1.58 (-3.19 to 0.03) | 0.055 |  |
| Baseline DLCO (MD, % predicted) | 1.85 (0.84 to 2.85) | <0.001 | | 1.83 (0.89 to 2.78) | <0.001 |  |
| FVC change (MD, % predicted) | 1.26 (0.85 to 1.68) | <0.001 | | 1.24 (0.79 to 1.69) | <0.001 |  |

Abbreviations: RR, risk ratio; HR, hazard ratio; MD, mean difference; DL, DerSimonian-Laird; REML, restricted maximum likelihood; CI, confidence interval; BMI, body mass index; FVC, forced vital capacity; DLCO, diffusing capacity for carbon monoxide.

**Supplementary Table 8** Leave-one-out sensitivity analyses for mortality outcomes.

| Study name | Effects with study removed | |
| --- | --- | --- |
|  | Effect (95% CI) | p-value |
| Mortality (RR) |  |  |
| None (main) | 0.91 (0.87 to 0.94) | <0.001 |
| Comes 2022 (CARE-PF) | 0.90 (0.87 to 0.94) | <0.001 |
| Comes 2022 (UCSF) | 0.91 (0.87 to 0.94) | <0.001 |
| Jouneau 2020 (nintedanib) | 0.90 (0.86 to 0.95) | <0.001 |
| Jouneau 2020 (placebo) | 0.90 (0.85 to 0.95) | <0.001 |
| Lee 2023 | 0.91 (0.88 to 0.94) | <0.001 |
| Sangani 2021 | 0.91 (0.87 to 0.94) | <0.001 |
| Yamaguchi 2022 | 0.91 (0.87 to 0.94) | <0.001 |
| Yoon 2024 | 0.86 (0.76 to 0.98) | 0.021 |
| Mortality (HR, BMI as continuous variable) |  |  |
| None (main) | 0.94 (0.92 to 0.96) | <0.001 |
| Alakhras 2007 | 0.95 (0.93 to 0.96) | <0.001 |
| Alhamad 2020 | 0.94 (0.92 to 0.96) | <0.001 |
| Aono 2020 | 0.94 (0.92 to 0.96) | <0.001 |
| Comes 2022 (CARE-PF) | 0.94 (0.92 to 0.96) | <0.001 |
| Comes 2022 (UCSF) | 0.93 (0.91 to 0.96) | <0.001 |
| Gao 2021 (SIPFR) | 0.94 (0.92 to 0.96) | <0.001 |
| Ikezoe 2017 | 0.94 (0.92 to 0.96) | <0.001 |
| Jouneau 2022 (2) | 0.94 (0.93 to 0.96) | <0.001 |
| Kishiaba 2021 | 0.94 (0.92 to 0.96) | <0.001 |
| Kono 2023 | 0.94 (0.92 to 0.96) | <0.001 |
| Li 2019 | 0.94 (0.92 to 0.96) | <0.001 |
| Snyder 2019 | 0.94 (0.92 to 0.96) | <0.001 |
| Suzuki 2018 | 0.94 (0.92 to 0.96) | <0.001 |
| Suzuki 2021 | 0.94 (0.92 to 0.96) | <0.001 |
| Suzuki 2021 (2) | 0.94 (0.92 to 0.96) | <0.001 |
| Yamazaki 2022 | 0.94 (0.92 to 0.96) | <0.001 |
| Yoon 2024 | 0.94 (0.92 to 0.95) | <0.001 |
| Zinellu 2021 | 0.94 (0.93 to 0.96) | <0.001 |
| Zinellu 2022 | 0.94 (0.93 to 0.96) | <0.001 |
| Mortality (HR, obese vs. non-obese) |  |  |
| None (main) | 0.75 (0.55 to 1.02) | 0.069 |
| Comes 2022 (CARE-PF) | 0.84 (0.64 to 1.11) | 0.218 |
| Comes 2022 (UCSF) | 0.75 (0.44 to 1.26) | 0.277 |
| Yoon 2024 | 0.66 (0.52 to 0.83) | <0.001 |

The row “None (main)” represents the primary meta-analysis including all eligible studies.

Abbreviations: RR, risk ratio; HR, hazard ratio; CI, confidence interval; BMI, body mass index; CARE-PF, Canadian Registry for Pulmonary Fibrosis; UCSF, ILD registry at the University of California, San Francisco; SIPFR, Swedish IPF Registry.

**Supplementary Table 9** Leave-one-out sensitivity analyses for hospitalization outcomes.

| Study name | Effects with study removed | |
| --- | --- | --- |
|  | HR (95% CI) | p-value |
| Hospitalization (univariable) |  |  |
| None (main) | 0.97 (0.95 to 0.99) | 0.013 |
| Jalaber 2021 | 0.97 (0.95 to 1.00) | 0.026 |
| Jouneau 2022 (2) | 0.98 (0.96 to 0.99) | 0.001 |
| Kim 2021 | 0.95 (0.90 to 1.00) | 0.044 |
| Yoon 2024 | 0.95 (0.89 to 1.02) | 0.138 |
| Hospitalization (multivariable) |  |  |
| None (main) | 0.98 (0.95 to 1.00) | 0.075 |
| Jouneau 2022 (2) | 0.99 (0.98 to 0.99) | <0.001 |
| Kim 2021 | 0.94 (0.85 to 1.04) | 0.240 |
| Yoon 2024 | 0.94 (0.85 to 1.05) | 0.279 |

The row “None (main)” represents the primary meta-analysis including all eligible studies.

Abbreviations: HR, hazard ratio; CI, confidence interval

**Supplementary Table 10** Leave-one-out sensitivity analyses for baseline lung function outcomes.

| Study name | Statistics with study removed | |
| --- | --- | --- |
|  | MD (95% CI) | p-value |
| Baseline FVC |  |  |
| None (main) | -1.61 (-3.10 to -0.12) | 0.034 |
| Alakhras 2007 | -1.81 (-3.33 to -0.29) | 0.020 |
| Comes 2022 (CARE-PF) | -1.44 (-3.20 to 0.31) | 0.108 |
| Comes 2022 (UCSF) | -1.35 (-3.00 to 0.30) | 0.109 |
| Jouneau 2020 (nintedanib) | -1.17 (-2.29 to -0.04) | 0.042 |
| Jouneau 2020 (placebo) | -1.67 (-3.28 to -0.06) | 0.042 |
| Jouneau 2022 (pirfenidone) | -1.56 (-3.31 to 0.19) | 0.081 |
| Jouneau 2022 (placebo) | -1.86 (-3.53 to -0.19) | 0.029 |
| Lee 2023 | -1.74 (-3.40 to -0.08) | 0.040 |
| Sangani 2021 | -1.93 (-3.35 to -0.50) | 0.008 |
| Yamaguchi 2022 | -1.59 (-3.10 to -0.07) | 0.040 |
| Baseline DLCO |  |  |
| NONE (main) | 1.85 (0.84 to 2.85) | <0.001 |
| Alakhras 2007 | 1.77 (0.73 to 2.82) | <0.001 |
| Comes 2022 (CARE-PF) | 2.07 (0.97 to 3.17) | <0.001 |
| Comes 2022 (UCSF) | 2.02 (0.92 to 3.11) | <0.001 |
| Jouneau 2020 (nintedanib) | 2.00 (0.90 to 3.10) | <0.001 |
| Jouneau 2020 (placebo) | 1.63 (0.63 to 2.63) | 0.001 |
| Jouneau 2022 (pirfenidone) | 1.89 (0.67 to 3.11) | 0.002 |
| Jouneau 2022 (placebo) | 1.91 (0.59 to 3.22) | 0.004 |
| Lee 2023 | 1.52 (0.72 to 2.31) | <0.001 |
| Sangani 2021 | 1.90 (0.85 to 2.95) | <0.001 |
| Yamaguchi 2022 | 1.85 (0.92 to 2.79) | <0.001 |

The row “None (main)” represents the primary meta-analysis including all eligible studies.

Abbreviations: MD, mean difference; CI, confidence interval; FVC, forced vital capacity; CARE-PF, Canadian Registry for Pulmonary Fibrosis; UCSF, ILD registry at the University of California, San Francisco; DLCO, diffusing capacity for carbon monoxide.

**Supplementary Table 11** Leave-one-out sensitivity analyses for FVC change outcome.

| Study name | Statistics with study removed | |
| --- | --- | --- |
|  | MD (95% CI) | p-value |
| NONE (main) | 1.26 (0.85 to 1.68) | <0.001 |
| Jouneau 2020 (nintedanib) | 1.30 (0.86 to 1.73) | <0.001 |
| Jouneau 2020 (placebo) | 1.23 (0.80 to 1.66) | <0.001 |
| Jouneau 2022 (pirfenidone) | 1.39 (0.93 to 1.86) | <0.001 |
| Jouneau 2022 (placebo) | 0.97 (0.39 to 1.54) | <0.001 |
| Lee 2023 | 1.33 (0.87 to 1.78) | <0.001 |

The row “None (main)” represents the primary meta-analysis including all eligible studies.

Abbreviations: MD, mean difference; CI, confidence interval.

**Supplementary Table 12** Sensitivity analyses of pooled hazard ratios for mortality restricted to studies with multivariable adjustment.

| Outcome | HR (95% CI) | p-value |
| --- | --- | --- |
| Mortality (main) | 0.94 (0.92 to 0.96) | <0.001 |
| Mortality (multivariable analysis only) | 0.94 (0.92 to 0.96) | <0.001 |

The main analysis included 19 studies. The multivariable-adjusted-only sensitivity analysis included 16 studies (Alakhras 2007; Alhamad 2020; Aono 2020; Comes 2022 [CARE-PF]; Comes 2022 [UCSF]; Jouneau 2022 [2]; Kono 2023; Li 2019; Snyder 2019; Suzuki 2018; Suzuki 2021; Suzuki 2021 [2]; Yamazaki 2022; Yoon 2024; Zinellu 2021; Zinellu 2022). Three studies (Gao 2021 [SIPFR], Ikezoe 2017, and Kishiba 2021) were excluded from this restriction because only univariable estimates were available.

Abbreviations: HR, hazard ratio; CI, confidence interval.

**Supplementary Table 13** Sensitivity analyses of pooled hazard ratios for hospitalization (univariable) restricted to studies with NOS ≥ 5.

| Outcome | HR (95% CI) | p-value |
| --- | --- | --- |
| Hospitalization (main) | 0.97 (0.95 to 0.99) | 0.01 |
| Hospitalization (NOS ≥ 5) | 0.97 (0.95 to 1.00) | 0.03 |

The main analysis included four studies. Restriction to studies with NOS ≥ 5 included 3 studies (Jouneau 2022 [2]; Kim 2021; Yoon 2024), with Jalaber 2021 excluded due to a NOS score < 5.

Abbreviations: HR, hazard ratio; CI, confidence interval; NOS, Newcastle-Ottawa Scale.

**Supplementary Table 14** Univariable meta-regression analyses for mortality (BMI as a continuous variable).

| Variable | Study number | β (SE) | *p*-value | Adjusted R^2^ (%) |
| --- | --- | --- | --- | --- |
| Region (Asian) | 19 | 0.020 (0.020) | 0.308 | 6.122 |
| Study period (after 2014) | 19 | 0.033 (0.022) | 0.128 | 29.050 |
| ILD subtype (IPF) | 19 | -0.008 (0.024) | 0.750 | <0.001 |
| Antifibrotics use | 19 | 0.013 (0.026) | 0.617 | 5.821 |
| Follow-up time, months | 10 | -0.001 (0.002) | 0.442 | <0.001 |
| Age | 14 | -0.001 (0.002) | 0.599 | <0.001 |
| NOS score | 19 | 0.027 (0.016) | 0.093 | 31.805 |

β values are presented on the log-HR scale. Adjusted R^2^ (%) indicates the percentage of between-study variance (τ²) explained by each covariate.

Abbreviations: BMI, body mass index; HR, hazard ratio; SE, standard error; NOS, Newcastle-Ottawa Scale; IPF, idiopathic pulmonary fibrosis.
